# Molecular Characterization of a Novel Rubodvirus Infecting Raspberries

**DOI:** 10.3390/v16071074

**Published:** 2024-07-03

**Authors:** Ondřej Lenz, Igor Koloniuk, Tatiana Sarkisová, Radek Čmejla, Lucie Valentová, Martina Rejlová, Jiří Sedlák, Dag-Ragnar Blystad, Bijaya Sapkota, Zhibo Hamborg, Jiunn Luh Tan, Rostislav Zemek, Přibylová Jaroslava, Jana Fránová

**Affiliations:** 1 Institute of Plant Molecular Biology, Biology Centre, Czech Academy of Sciences, 370 05 Ceske Budejovice, Czech Republic; koloniuk@umbr.cas.cz (I.K.); sarkisova@umbr.cas.cz (T.S.); pribyl@umbr.cas.cz (P.J.); jana@umbr.cas.cz (J.F.); 2 Research and Breeding Institute of Pomology Holovousy Ltd., 508 01 Horice, Czech Republic; radek.cmejla@vsuo.cz (R.Č.); valentova@vsuo.cz (L.V.); martina.rejlova@vsuo.cz (M.R.); jiri.sedlak@vsuo.cz (J.S.); 3 Norwegian Institute of Bioeconomy Research, 1433 Aas, Norway; dag-ragnar.blystad@nibio.no (D.-R.B.); bijaya.sapkota@nibio.no (B.S.); zhibo.hamborg@nibio.no (Z.H.); 4 Faculty of Science, University of South Bohemia, 370 05 Ceske Budejovice, Czech Republic; jiunnluh@gmail.com (J.L.T.); rosta@entu.cas.cz (R.Z.); 5 Institute of Entomology, Biology Centre, Czech Academy of Sciences, 370 05 Ceske Budejovice, Czech Republic

**Keywords:** rubodvirus, raspberry, Rubus, HTS, aphids

## Abstract

A novel negative-sense single-stranded RNA virus showing genetic similarity to viruses of the genus *Rubodvirus* has been found in raspberry plants in the Czech Republic and has tentatively been named raspberry rubodvirus 1 (RaRV1). Phylogenetic analysis confirmed its clustering within the group, albeit distantly related to other members. A screening of 679 plant and 168 arthropod samples from the Czech Republic and Norway revealed RaRV1 in 10 raspberry shrubs, one batch of *Aphis idaei*, and one individual of *Orius minutus*. Furthermore, a distinct isolate of this virus was found, sharing 95% amino acid identity in both the full nucleoprotein and partial sequence of the RNA-dependent RNA polymerase gene sequences, meeting the species demarcation criteria. This discovery marks the first reported instance of a rubodvirus infecting raspberry plants. Although transmission experiments under experimental conditions were unsuccessful, positive detection of the virus in some insects suggests their potential role as vectors for the virus.

## 1. Introduction

Genus *Rubodvirus* (family *Phenuiviridae*) has been described relatively recently [1]. It includes four distinct species: *Rubodvirus argentinaense* (formerly grapevine Muscat rose virus, GMRV), *Rubodvirus armeniaense* (formerly grapevine Garan dmak virus, GGDV), *Rubodvirus mali* (formerly apple rubbery wood virus 1, ARWV1), and *Rubodvirus prosserense* (formerly apple rubbery wood virus 2, ARWV2). In 2022, a novel putative rubodvirus or rubodvirus-related species named qingdao RNA virus 3 (QRV3) was described [2].

The genome of rubodviruses consists of three genomic segments of negative-sense single-stranded RNA, (-)ssRNA, encoding the L-protein (RNA-directed RNA polymerase; RdRP), nucleocapsid protein (NP), and movement protein (MP). For QRV3, only segments of RdRP and NP have been published. In addition, the presence of two distinct versions of MPs and/or NPs associated with one specimen were reported for a few isolates of ARWV2 [1,3,4].

Within the family *Phenuiviridae*, rubodviruses represent one of five genera, which are known to infect plant hosts, either exclusively or in conjunction with insects: *Coguvirus*, *Laulavirus*, *Mechlorovirus*, *Rubodvirus*, and *Tenuivirus* [5]. Based on sequences of RdRP and NP, coguviruses and laulaviruses were identified as the closest relatives of rubodviruses [6]. Both coguviruses and laulaviruses encode the same three proteins as rubodviruses: RdRP, NP, and MP. While in laulaviruses each protein is encoded on a separate segment [7] like in tripartite rubodviruses, the genome of coguviruses is bipartite and one of its segments harbors both NP and MP genes in ambisense orientation [8]. Tenuiviruses encode one more non-structural protein on the fourth genome segment [9], and the genus *Mechlorovirus* possesses eight different genomic segments [10].

Rubodviruses have been reported from various countries worldwide [11,12,13,14,15,16], primarily infecting apples [1], pears [1,3], grapevines [6,15], and vegetable plants [2]. However, the specific impact of these viruses on plant health and disease symptoms of the other plants remains elusive, because they have so far been found mostly in mixed infections together with other viruses and/or viroids [1,6,15,17]. The only exception is apple rubbery wood disease, for which ARWV1 and ARWV2 have been established as causative agents [1]. Furthermore, it is hypothesised that symptom expression or severity of the disease could be influenced also by the presence of other viruses [1], as already documented for mixed infection of several viral species [18,19]. To date, the transmission of rubodviruses has been reported only by grafting, with no evidence of insect vectors [1,6].

In this study, we report the complete genome sequence of a new rubodvirus infecting raspberry plants, tentatively named raspberry rubodvirus 1 (RaRV1). The goal of this study was to molecularly characterise this virus, its variability, and its prevalence in raspberry plants and different insect species, with a specific focus on the potential of *Aphis idaei* to vector RaRV1 under controlled experimental conditions.

## 2. Materials and Methods

### 2.1. Plant/Insect Samples and RNA Isolation

Plant samples (preferentially symptomatic leaves) from wild, garden, and commercial raspberry plants from the Czech Republic (*n* = 483) and Norway (*n* = 196) were collected in 2021–2023 (Table S1). Arthropods (*n* = 168) found on the tested shrubs in the Czech Republic or from transmission experiments were also processed. Outdoor arthropod samples were collected directly with part of the plant and stored in sealed bags at 10 °C until processing (up to few days).

For total RNA isolation from samples of Czech origin, approximately 100 mg of fresh or frozen (−20 °C) young raspberry plant leaves, along with the whole bodies of aphids and small invertebrates or the heads and thoraxes of bigger insects, were subjected to a RiboSpin Plant Kit (GeneAll Biotechnology, Seoul, Republic of Korea) following the manufacturer’s instructions with DNase digestion step on column.

For samples originating from Norway (plant samples only), the RNA extractions were performed using a Norgen Plant/Fungi RNA Kit (Norgen Biotek Corp, Thorold, Canada) according to the manufacturer’s instructions, with some modifications. The quantity of RNA was assessed using a NanoDrop spectrophotometer (NanoDrop Technologies, Wilmington, DE, USA).

The extracted RNA from both the plant and insect samples was stored at −80 °C for future use.

### 2.2. RT–PCR Screening and Sanger Sequencing of Plant and Insect Samples

Reverse transcription of the Czech samples was carried out using random primers, M-MLV Reverse Transcriptase (Invitrogen, Waltham, MA, USA), and 0.5 µg of plant RNA or less from insects; these were used as a template for each 20 µL reaction. PCR with RaRV1 detection primers 3125 + 3128 corresponding to the NP gene was performed using PPP-Master Mix (TopBio, Vestec, Czech Republic) under the following conditions: 94 °C for 1 min, 35× (94 °C for 15 s, 60 °C for 30 s, 72 °C for 45 s), and 72 °C for 10 min. 

Complementary DNA synthesis of the Norwegian samples was performed with random primers using SuperScript IV Reverse Transcriptase (Invitrogen) with 1 µg of RNA as a template. PCR was carried out as described above.

All the amplicons were purified either from 1% agarose gel or directly from the PCR mixture using the Expin Combo GP mini (GeneAll). The purified amplicons were sequenced in both directions (Eurofins Genomics, Ebersberg, Germany) and identified using the BLAST service of the NCBI.

The presence of nine other viruses—namely, the black raspberry necrosis virus (BRNV), raspberry enamovirus 1 (RaEV1), raspberry bushy dwarf virus (RBDV), raspberry leaf blotch virus (RLBV), raspberry leaf mottle virus (RLMV), raspberry ringspot virus (RpRSV), raspberry vein chlorosis virus (RVCV), Rubus yellow net virus (RYNV), and strawberry latent ringspot virus (SLRSV)—was evaluated by RT–PCR of RaRV1-positive samples with appropriate primers and conditions (Table S2—[20,21,22,23,24,25,26]. For positive insect samples, detection of eventual plant diet was performed by using the primers AtropaNad2.1a and AtropaNad2.2b, targeting the *ndhB* gene (Table S2—[27]). Selected amplicons were purified from a 1% agarose gel and Sanger-sequenced as described above.

### 2.3. High Throughput Sequencing and Analysis of the V2 Sample

In June 2021, leaves from *Rubus idaeus* cv. Canby showing symptoms of vein clearing, mosaicism, and leaf curl were collected in Volanice, Eastern Bohemia (isolate V2). The total RNA was isolated from sample V2 as above; its quantity and quality were checked by Qubit HS RNA and IQ assays (Invitrogen, Waltham, MA, USA). A sequencing library was prepared with a NEBNext Ultra II Directional RNA Library Prep kit in combination with NEBNext Multiplex Oligos (NEB, Ipswich, MA, USA) that were processed on the NovaSeq 6000 platform (Admera Health Biopharma Services, New York, NY, USA) using a paired-end (2 × 150b) configuration.

The obtained paired-end reads were first prefiltered by mapping onto the genome of *Rubus idaeus* cv. Malling Jewel (Acc. Nos. CM057965-CM057971), the *Rubus chingi* mitochondrial genome (ON478176), and the *Rubus biflorus* chloroplast genome (NC_080344). The remaining unmapped reads were then de novo assembled (Geneious assembler), and the resulting contigs were compared to local BLAST [28] databases of viral nucleotide (using blastn, e-value cut-off = 0.05) and protein sequences (using blastx, e-value cut-off = 0.05), established from GenBank sequences available in May 2023.

### 2.4. Sanger Sequencing of V2 Isolate of Raspberry Rubodvirus 1 (RaRV1)

Primers for the PCR amplification and Sanger sequencing of the whole genome of putative novel rubodvirus were designed according to assembled contigs (Table S2). Virus-specific cDNA was prepared using M-MLV Reverse Transcriptase (Invitrogen) and primers targeting genomic RNA. Overlapping fragments covering all the HTS contigs of putative rubodvirus were amplified using PPP-Master Mix (TopBio, Vestec, Czech Republic) and appropriate primers under following conditions: 94 °C for 1 min, 35× (94 °C for 30 s, 60 °C for 30 s, 72 °C for 45 s), and 72 °C for 10 min. For 5′/3′-RACE, cDNAs prepared by specific reverse/forward primers (Table S2) were amplified by a homopolymeric tail (dC or dA) by terminal deoxynucleotidyl transferase (Invitrogen) or *E. coli* poly(A) polymerase (New England Biolabs, Ipswich, MA, USA), respectively, and amplified using the corresponding primers. All amplicons were analysed on a 1% agarose gel, excised, purified (Gel and PCR CleanUp kit, Macherey-Nagel, and Expin Combo GP mini, Gene All), and Sanger-sequenced (Eurofins Genomics).

### 2.5. Transmission Experiments

All experiments were conducted in air-conditioned glasshouse chambers at a temperature of 18 °C and a 16 h light to 8 h dark photoperiod. Virus-free colonies of *Aphis idaei* (individual lines from single eggs) were reared on virus-free raspberry rooted from tissue culture plants in aphid mesh chambers (18 °C, 75% humidity, 16 h light to 8 h dark period). Before the experiment, *Aphis idaei* aphids were reared on *Rubus idaeus* cv. Tulameen (TUP) plants propagated from tissue cultures and confirmed to be free of viruses through HTS. Two TUP plants (approximately 10 cm tall) rooted from tissue cultures and one *Nicotiana occidentalis* 37B plant grown from seeds were used as recipient plants for transmission.

Adult aphids were first transferred to Petri dishes to starve for one hour and subsequently transferred to leaves from the donor plant *R. idaeus* cv. Malling Jewel (isolate H1), which exhibited leaf curling symptoms and tested positive for RaRV1, RBDV, and RLMV by RT–PCR. After feeding on donor plant leaves for 96 h (acquisition period), the aphids were transferred onto two *R. idaeus* recipient plants (20 and 40 aphid individuals) and one plant of *N. occidentalis* 37B (20 individuals). After 20 weeks, the aphids living on the raspberry recipient plants were killed by insecticide (FAST M, AgroBio, Opava, Czech Republic, active ingredient: deltamethrin 0.12 g/L) applied at the recommended dose via a hand sprayer. All recipient plants and aphid samples (*n* = 5) were tested for the presence of RaRV1 and RLMV 3, 4, 8, and 20 weeks after the start of transmission, with plants still being tested at week 32. The plants and aphids were not tested for RBDV due to its non-transmissibility to insects. Additionally, selected groups of aphids (*n* = 5) were tested 48 and 96 h after the start of feeding on donor plants (cv. Malling Jewel, H1). For this test, random-primed cDNA prepared from total RNA was used.

### 2.6. Data Analyses

The HTS reads were trimmed and end-paired in CLC Genomics Workbench 9.5.1 (Qiagen, Hilden, Germany), contigs were assembled in Geneious R11 software, version 11.1.5 (Biomatters, Auckland, New Zealand), using integrated Geneious assembler. Open reading frames (ORFs) were identified via ORFfinder and BLASTp comparisons. Transmembrane domains were tested in the online versions of DeepTMHMM (version 1.0.24, https://dtu.biolib.com/DeepTMHMM, accessed on 15 February 2024, [29]) and CCTOP (version s.1.1.0, https://cctop.ttk.hu/, accessed on 15 February 2024, [30]), and these and other domains were searched by PSI Pred (version 4.0, http://bioinf.cs.ucl.ac.uk/psipred, accessed on 15 February 2024) and InterPro (version 98.0, https://www.ebi.ac.uk/interpro/, accessed on 15 February 2024, [31]). Secondary RNA structures were assessed by RNAfold, version 2.4.17 [32].

Multiple alignments were carried out in Geneious R11 software using the MUSCLE and MAFFT algorithms, and phylogenetic trees were constructed with Geneious Tree Builder using the Jukes–Cantor neighbour-joining method with 500 bootstrap replicates. Phylogenetic trees were visualised via iTOL [33].

## 3. Results

### 3.1. HTS and Sanger Sequencing of the V2 Sample

Sequencing output of the V2 sample yielded a total of 23.8 million trimmed paired-end HTS reads, which were prefiltered by mapping onto plant nuclear, mitochondrial, and chloroplast genomes (see Section 2.2). The remaining unmapped 608,470 reads were de novo assembled into 87 686 contigs, from which 3176 were longer than 500 nt.

In addition to several contigs exhibiting similarity to isolates of raspberry bushy dwarf virus (RBDV; 93.3–98.6% nt identity, aa identity 98.6–100%) and contigs of plant origin, which were not eliminated during the prefiltering process, four contigs shared high similarity with the apple rubbery wood virus 1 (ARWV1) and apple rubbery wood virus 2 (ARWV2); selected statistics are shown in Table 1. BLASTn analyses of all other contigs limited to the genus *Rubodvirus* in the local database did not reveal any other significant hits. HTS data from this experiment were deposited in the NCBI SRA storage repository under BioProjectID PRJNA1028176—experiment SRX24439621

The genome sequences of all four segments of the putative novel rubodvirus were validated with Sanger sequencing and completed by the 5′/3′-RACE approach, and the sequences were deposited in GenBank (acc. nums. PP732065-PP732068).

Further RT–PCR and Sanger sequencing of the V2 sample confirmed the presence of RBDV and revealed the presence of RVCV with a nucleotide identity of 92.3% to the GenBank complete genome MK240091. This virus was not captured by the HTS of the original sample using the described approach. Subsequent backward mapping of all the 23.8 million paired-end reads onto the RVCV MK240091 reference produced only 21, usually separated, hits (86.8 to 95.9% nt identity for each).

### 3.2. Genome of RaRV1—Isolate V2

**Figure 1:**
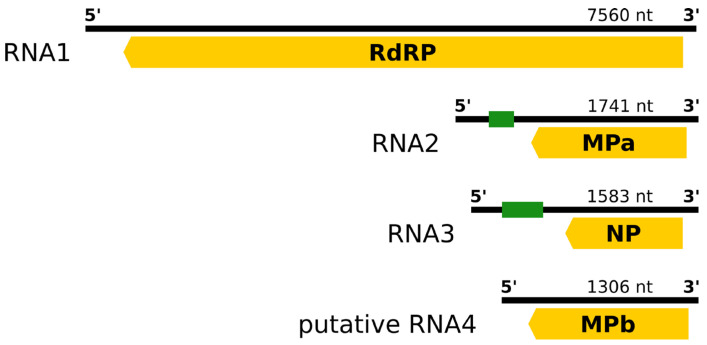
Schematic representation of the raspberry rubodvirus 1 (RaRV1) genome. Green rectangles on RNA2 and RNA3 depict U-rich regions of imperfect tandem repeats (G/N)1-3(U)3-7; yellow rectangle arrows represent particular ORFs (arrow depicts the orientation).

Sample V2 (Volanice) contained four segments, each bearing one ORF with a long 5’UTR—especially in the case of RNA2 (377 nt) and RNA3 (644 nt)—and inverted repeats at the ends of the genome (Figure 1). The 5’UTRs of RNA2 and RNA3 harbour U-rich regions of imperfect tandem repeats consisting of blocks of 3–7 uridines separated by 1–2 other nucleotides, often guanosine.

On the first three segments, analyses identified the RdRP (RNA1), movement protein (RNA2), and capsid protein (RNA3) domains. Furthermore, all three ORFs exhibited similarity to the corresponding proteins of rubodviruses (Table 2 and Table S3).

However, no known functional domain or transmembrane region was found on the ORF4 putative product. Due to its relative similarity to the movement protein encoded on ORF2 (48.8% nt identity, 19.0% aa identity), movement proteins of other rubodviruses (Table 2 and Table S3), and clustering with MPs of rubodviruses on a phylogenetic tree (see below), ORF4 was preliminarily designated MPb. Further analyses revealed that the identity between RaRV1 MPa and MPb (31.2% nt, 19.0% aa) was also considerably lower than the identities between previously reported versions of MPb and their corresponding MPa sequences in rubodviruses (65.3–66.0% nt identity; 66.6–66.8% aa identity).

### 3.3. Genome Termini and Putative Secondary Structures

**Figure 2:**
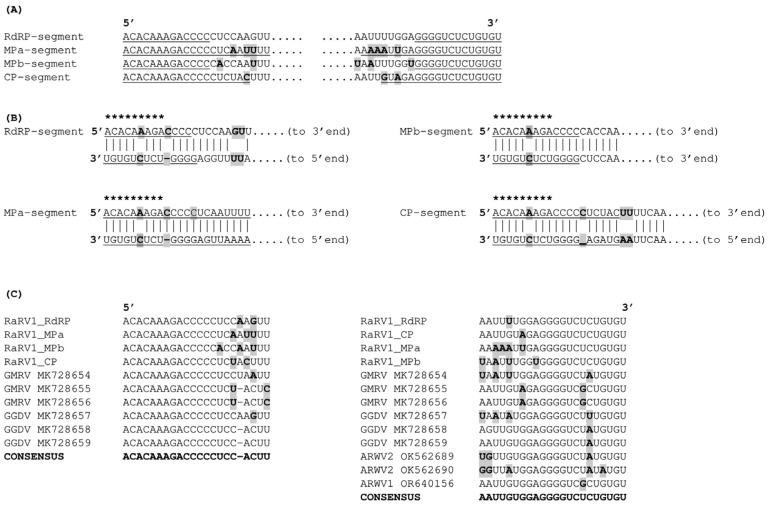
5′UTR and 3′UTR terminal sequences of the raspberry rubodvirus 1 (RaRV1). (**A**) Conservation of inverted repeats at the ends of vRNAs of RaRV1; bases predicted by RNAfold software to participate in end-to-end pairing are underlined; (**B**) Complementarity of IR at the 5′ and 3′ ends of the same segment; bases predicted by RNAfold software to participate in end-to-end pairing are underlined (the same as in (**A**)); bases of the 5′ end previously reported in other bunyavirids to form nonpairing hooks bound to RdRP are marked by asterisks above the sequence; (**C**) Alignment of the 5′ and 3′ ends of different available rubodviruses. All mismatches from consensus sequence in (**A**–**C**) are in bold and shaded in grey.

The 5′ and 3′ terminal sequences of RaRV1 (22 nt) were conserved, both between segments (Figure 2A) and within other rubodviruses (Figure 2C). They were also highly reverse-complementary within each segment (Figure 2B), creating imperfect inverted repeats (IRs). RNAfold software predicted the formation of pseudocircularised panhandles of each genome segment due to pairing between the majority of the bases of the 5′ and 3′ IRs (Figure 2A,B). Similarly to other rubodviruses, there was a one-base deletion at the 10th position of the alignment of the 5′ and 3′ IRs in all of the RaRV1 segments (Figure 2B).

**Figure 3:**
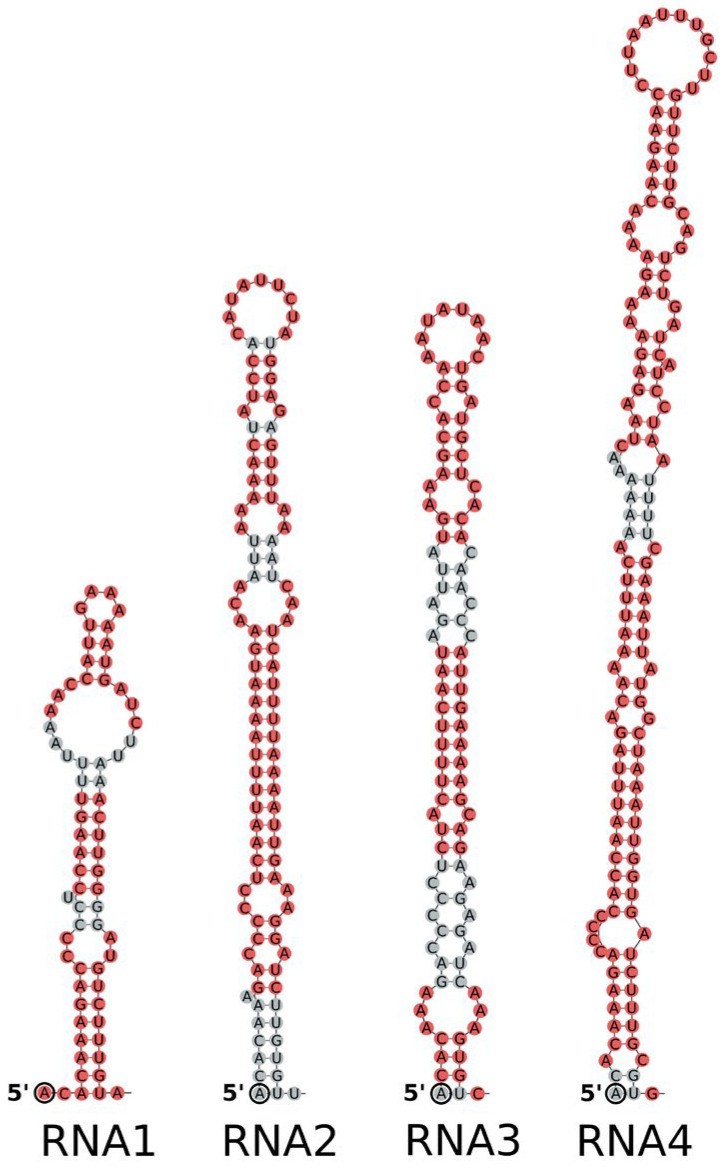
Stem-loop structures predicted for the 5′ ends of the raspberry rubodvirus 1 (RaRV1) viral RNAs when 5′UTRs were analysed separately. No G–U pairs were allowed in the analysis, and the structures predicted by RNAfold were redrawn in Inkscape; the structures with more than 75% predicted probability are in red; the terminal 5′ base of each segment is in a circle.

When the 3’-UTR was excluded from the analysis and the folding of the 5′-UTR sequence was analysed independently, RNAfold showed high-probability stem-loop structures within the first 70 nt of all viral RNAs (Figure 3). The formation of these structures was enabled by the presence of 4–15 nt long IRs in this region, although the sequence of a particular IR differed among segments. Notably, the predicted stem-loop structures did not include a U-rich region and lacked any of the conserved transcription termination signals (TTSs) reported previously for similar terminal structures of coguviruses and phenuiviruses [8,34,35]. Furthermore, our analysis did not reveal any other conserved TTS motifs across the entire 5′UTR of any of the four RaRV1 segments.

### 3.4. Phylogeny

**Figure 4:**
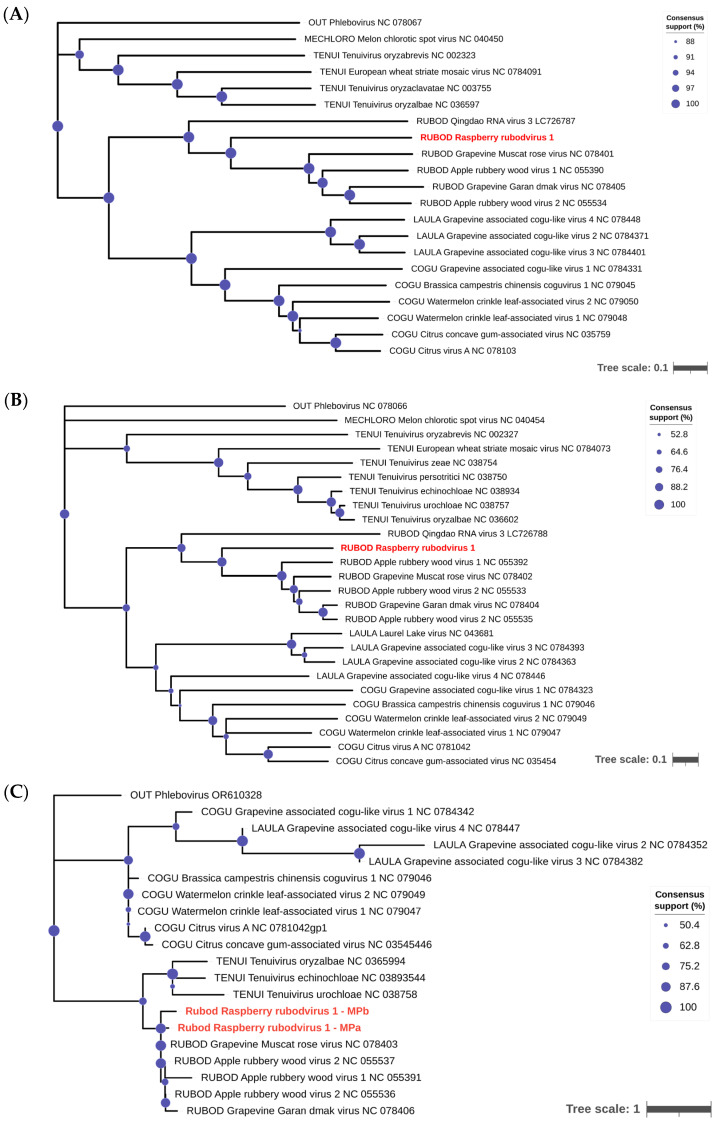
Phylogenetic trees of selected plant phenuivirids and the raspberry rubodvirus 1 (RaRV1) based on amino acid sequences of RdRP (**A**), NP (**B**), and MP (**C**). All available reference sequences for plant phenuivirids were chosen; as an outgroup, selected phlebovirus sequences were used, RaRV1 sequences obtained are in red. The scale bar represents substitutions per site.

Alignments of the nucleotide and amino acid sequences of the putative proteins revealed that RaRV1, similarly to QRV3, was more distantly related to the other rubodviruses. The average interspecies distances between proteins of ARWV1, ARWV2, GGDV, and GMRV ranged from 49.8 to 90.6% in aa identity (49.8–61.6% for RdRP, 67.6–90.6% for NP, 54.9–81.5% for MPa, and 55.4–81.7% for MPb, if present); however, the maximum aa identity of RaRV1 proteins to this group was only 45.3% (Table 2 and Table S3). In phylogenetic trees of selected plant phenuivirids based on RdRP, MP, and NP, all of the rubodviruses clustered into a monophyletic group, although RaRV1 (and QRV3) was more distant (Figure 4). Interestingly, the MPs of the rubodviruses shared the closest ancestor with those of the tenuiviruses (Figure 4C), while among the RdRP and NP, those of the rubodviruses were more closely related to those of the cogu- and laulaviruses.

### 3.5. Prevalence of RaRV1

Out of the 679 screened plants (Table S1), there were only 10 RaRV1-positive samples, originating from production fields and gardens in the East Bohemia part of the Czech Republic. In all these samples, RaRV1 was present in a mixed infection with the other viruses (Table 3).

**Figure 5:**
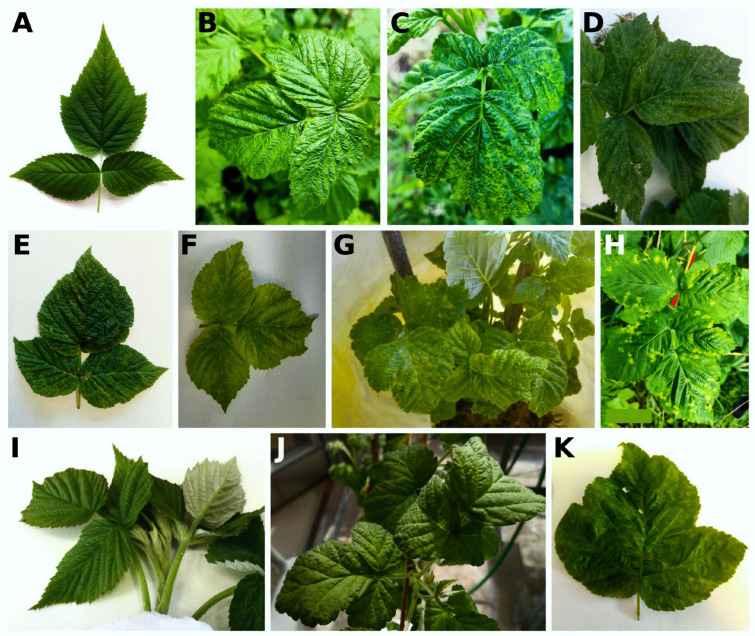
Symptomatic raspberry plants infected with the raspberry rubodvirus 1 (RaRV1) and healthy control. (**A**) cv. Tulameen, B730—healthy control; (**B**) V2 (B350)—vein clearing, mosaic, leaf curl; (**C**) V4 (A805)—vein clearing, mosaic, leaf curl; (**D**) V5 (A806)—vein clearing, yellowing; (**E**) MC1 (A986)—vein clearing, yellowing; (**F**) MC2 (CG757)—vein clearing, yellowing, necrosis; (**G**) CH525 (B631, B632)—vein clearing, mosaic; (**H**) NB1 (A807)—yellow blotches, leaf malformation, (**I**) D23 (B315)—no symptoms; (**J**) H1 (B467)—leaf curling; (**K**) O4M (CF863)—vein clearing, yellowing.

Symptoms of vein clearing were observed on the leaves of seven plants, whereas in six plants, RVCV was present, among other viruses. Raspberry bush cv. Bulharský rubín (isolate NB1, which was positive for RaEV1, RBDV, RLBV, and RVCV) displayed severe yellow blotches on its leaves, which is a common symptom associated with the presence of RLBV (Figure 5). Both leaf curling and no symptoms were observed on the shrubs cv. Malling Jewel (isolate H1) and cv. Canby (isolate D23), respectively, in which neither RVCV nor RLBV were detected. However, there were only young shoots available for cv. Canby, in which BRNV, RBDV, RLMV, and RaRV1 were present without showing viral symptoms at the time of sampling (16 May 2022, Figure 5).

In addition to the plant samples, RaRV1 was also detected and confirmed by Sanger sequencing in 2 insect samples (out of the 168 tested). These were collected from the RaRV1-positive plant V4, the minute pirate bug *Orius minutus* L. (Hemiptera: Anthocoridae; sample A801), and one batch (*n* = 5) of small raspberry aphids, *Aphis idaei* van der Goot (Homoptera: Aphididae; sample A803). Both insect samples were also positive for RVCV (confirmed by Sanger sequencing) and negative for plant debris (primers AtropaNad2.1a and AtropaNad2.2b). The V4 plant was tested twice (in 2021 and 2022) and was always positive for RaRV1, RVCV, and RBDV.

### 3.6. Variability of RaRV1 Isolates

In the CH525 plant, Sanger sequencing revealed a divergent sequence of RaRV1 (isolate CH525), substantially different from that of the original V2. Using the same primers as for the Sanger sequencing of the V2 isolate (Table S2), two longer fragments of the CH525 genome were obtained: an almost complete sequence of the NP segment (1477 nt, including the complete NP-ORF; acc. no. PP732069) and a partial sequence of the RdRP-ORF (1186 nt; acc. no. PP732070).

**Figure 6:**
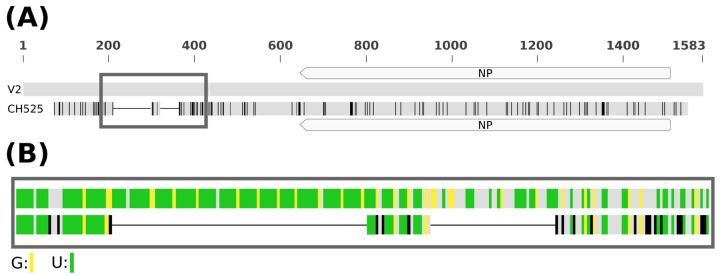
Comparison of U-rich regions between the V2 and CH525 isolates of raspberry rubodvirus 1 (RaRV1). (**A**) Position of U-rich regions on both sequences (in rectangle), (**B**) more detailed view of U-rich regions with highlighted uridine (green) and guanosine (yellow) nucleotides. In both schemes, nucleotides of CH525 isolate differing from those of V2 isolate are marked black.

A comparison with the V2 isolate revealed 95.8% aa identity in the complete NP sequence and 94.8% aa identity in the partial RdRP sequence (404 out of 2476 aa). Furthermore, the 5’-UTR of the CH525 isolate lacked a substantial part (131 nt) of the U-rich region present in the V2 genome (Figure 6). In total, 12 of the 18 original imperfect tandem repeats were missing. The other primers used in the sequencing of the V2 isolate did not yield any PCR products, suggesting that the rest of the sequences might also be different.

To elucidate if the RaRV1 genome indeed consists of four segments, we inspected the presence and variability of MPa and MPb segments in other RaRV1-positive samples using the same primer combination as for the V2-isolate sequencing. All detected versions of MP (Table 4) shared almost the same sequence as the corresponding version of V2isolate MP (up to three nucleotide differences), leading to at most 1–2 amino acid changes (with the exception of the NB1 isolate, in which a nucleotide insertion introduced premature STOP-codon). Six isolates from three different localities (Volanice, Míčov, Ostroměř) possessed both MPa and MPb, while in the rest of the isolates, only one version of MP was detected by primers designed according to the V2-isolate sequence (Table 4).

### 3.7. Aphid Transmission Experiments

Three groups of *Aphis idaei* (20, 20, and 40 individuals) were tested to transfer RaRV1 (and RLMV) to one plant of *N. occidentalis* and two plants of *R. idaeus* after 96 h of feeding on RaRV1- and RLMV-positive plants. The aphids on the *N. occidentalis* recipient plant died 4 days after transfer, the aphids on the *R. idaeus* recipient plants were allowed to colonise the plants for another five months. None of the three recipient plants developed any symptoms, and all of them remained negative for RaRV1 and RLMV throughout the whole monitored period (3–32 weeks). Aphid samples taken from recipient raspberry plants at the same time intervals as the plants were also always negative for both viruses. The selected groups of aphids that were tested directly after 48 and 96 h of feeding on the donor plant were also free of both tested viruses.

## 4. Discussion

In this study, we report a virus that represents a new species of the genus *Rubodvirus* (family *Phenuiviridae*), tentatively named raspberry rubodvirus 1 (RaRV1). Its genome is divided into 3–4negative ssRNA segments, each bearing one ORF. The first three ORFs are similar to the respective ORFs of the other rubodviruses, which encode the L-protein (RdRP), NP, and MP, clearly placing RaRV1 within the rubodvirus group.

However, an important question is if the fourth segment is part of a single isolate—the virus would thus be quadripartite—or if the fourth segment belongs to another co-infecting rubovirus or is a remnant of some reassortment. Although different variants of MP and/or N-protein segments in one host have already been reported for ARWV-2 rubodvirus [1,3,4], the variants reported so far were much more similar to each other in both the 5′UTR and protein sequence (over 66% aa sequence identity) and possessed recognizable functional domains (i.e., plant virus MP or NP of Phenuiviridae, respectively). In the case of RaRV1, the similarity of MPb to MPa was significantly lower (19% aa identity), the nucleotide sequence of the 5′UTR of both segments differed substantially, and comparison with the databases did not reveal any known functional domain. If it was an MP from another isolate of rubovirus, we did not find any other fragments from such a rubodvirus in the NGS data (isolate V2). Moreover, we detected the same configuration of very similar MPs in another six isolates from three different locations. That could be explained either by co-infection with two viral strains possessing together the exact combination of MPs (one strain MPa, second strain MPb) at different localities or by the fact that MPb function as fourth segment of RaRV1. If the latter were true, the failure to detect one or both MPs (using primers designed for the V2 isolate) in the other six samples could be explained by the presence of different MP version(s). Although a definitive decision cannot be made now, we currently designate the MPb-coding fragment as “putative RNA4”. It is apparent that the function of this particular protein may be different and has yet to be investigated.

Like those of other rubodviruses (and all members of the order *Bunyavirales*), the genomic segments of RaRV1 possess terminal sequences that are reverse-complementary to each other within a particular segment. In the case of RaRV1, they are 22 nt long, and a major part of them was predicted to form a panhandle structure. However, experimental studies revealed that only the second half of these sequences paired together (creating a so-called “distal duplex”), as the first 10 nucleotides of the 5′ end, are bound to the L-protein (RdRP) either in the virion/nucleoprotein or at the time of replication initiation [36]. This might be the reason why all 3′ ends of rubodviruses, including those of RaRV1, have two mismatches (including one deletion) in the first 10 bases of the 5′ end of each genome segment (Figure 2B). A similar “gap” in the 3′ end sequence can also be found on some segments of other plant phenuivirids (e.g., RNA1 of coguviruses [3,8,34,37]), but not all of them. However, whether this feature is important for rubodviruses or plant phenuivirids remains to be elucidated.

Transcription of most genes from *Bunyavirales* is terminated at specific sites before the very 5′ end of the genome, and only some transcripts are transcribed to the end of the template [36]. Usually, there are specific, few-nucleotides-long transcription termination sequences (TTSs) within secondary structures [8,34,35]. In the case of RaRV1, we were not able to find such TTS motifs conserved in all four (three) segments; however, secondary structures were predicted at the 5′ end of the genome. As these structures also consist of 5′ end IRs, they can be created by the elongation of mRNA at the time, when the 5′ end is not bound to the L-protein (RdRP) and does not form a distal duplex with the 3′ end of the genome (now occupied by the originating mRNA). However, the real function of the predicted secondary structures in transcription termination remains to be experimentally proven.

Among the 10 RaRV1 isolates found on raspberry plants, one (CH525) substantially differed. An attempt to obtain its full sequence using V2-specific primers yielded an almost complete sequence of its NP segment and a partial sequence of the RdRP segment. A comparison of protein sequences revealed identity on the species demarcation border (95% aa identity in any gene). However, as the rest of the protein sequences were not available, we cannot reliably conclude that the CH525 isolate belongs to the RaRV1 species. There can be more variability in the rest of its genome, as suggested by a significant deletion in the 5’UTR of RNA3 (encoding NP) and by the fact that we were not able to amplify any other fragment using other primers for the resequencing of the V2 isolate. The CH525 isolate can thus be a member of a new species of the *Rubodvirus* genus closely related to RaRV1. The retention of some tandem repeats in the U-rich region and part of the middle of the deletion (Figure 6) might further suggest the importance of these sequences for this segment of the RaRV1 genome (the repetitions are not present on segments encoding RdRP and MPb). The fact that we were not able to detect any of the MP variants even in the D23 isolate further supports more variability among segments of RaRV1 genome. 

In all 10 RaRV1-positive plants, the virus was found in mixed infection with the other viruses (Table 3). Nine infected plants were symptomatic, and only one young sprout was symptomless. However, this latter plant could have developed symptoms later in the season. As attempts to transmit the virus were unsuccessful, it cannot be proven if the virus alone is able to produce any symptoms or if the symptoms observed are the result of infection of the other viruses and/or their combination. This finding is similar to that of other reported rubodviruses, which are usually found in mixed infections with other viruses and/or viroids [1,6,15,17].

The screening of the arthropods colonizing the infected plants revealed the presence of RaRV1 in one batch of small raspberry aphids (*A. idaei*) and one individual of predatory bug specimen (*O. minutus*). At the same time, the plant diet as a source of the virus was excluded. Although transmission was not proved experimentally and the presence of RaRV1 in insects collected in nature was rare, the possibility of the eventual nonpropagative acquisition of the virus by arthropods cannot be excluded. *Aphis idaei* colonises plants naturally, and *O. minutus* can possibly transfer the virus by occasional phytophagy, which has already been documented for *Orius* species [38,39]. Possible acquisition of the virus by aphids can also be influenced/enabled by the presence of RVCV, which is transmitted by *A. idaei* in a persistent propagative manner [40] and which was detected in both RaRV1-positive insect samples in natura.

To our knowledge, RaRV1 represents the first rubodvirus described in raspberry plants. This species putatively possesses a fourth genomic segment, which encodes a protein distantly related to MP with currently undetermined function. Our analyses revealed variability of the virus, its prevalence in raspberry plants, and its presence in certain insect species. These findings raise questions for further experimental research, particularly regarding the true function of MPb and the frequency and mechanism of potential transmission by insects.

## Figures and Tables

**Table 1:** Selected assembly and blastx statistics of the raspberry rubodvirus 1 (RaRV1) contigs. A single blastx hit from GenBank with the lowest e-value is listed for each contig.

Segment	Contig			Maximal Blastx Hits			
	Length (nt)	Number of Reads	Coverage (Reads/Base)	Virus	Accession	Identity (%)	Coverage (%)
RNA1	7728	4612	89.4	ARWV2	AWC67514	37.6	83.3
RNA2	1755	1758	150.3	ARWV2	UOA05155	50.9	45.3
RNA3	1571	5314	504.3	ARWV2	UOA05158	45.8	52.7
RNA4	1295	1434	165.3	ARWV2	WBB27576	23.9	65.8

**Table 2:** Comparison of raspberry rubodvirus 1 (RaRV1) protein-coding sequences to those of other rubodvirus species and rubodvirus-related qingdao RNA virus 3 (QRV3). Only the highest aa identity (%) for all available complete sequences of a particular species is listed, the highest values for a particular protein are in bold; n.a. = not analysed.

Virus	RdRP (2476 aa)	MPa (437 aa)	NP (287 aa)	MPb ^1^ (359 aa)
ARWV1	**34.5**	36.5	42.6	19.1
ARWV2	34.2	**36.6**	**45.3**	**19.7**
GGDV	31.6	34.5	41.2	18.2
GMRV	31.1	35.2	42.5	18.4
QRV3	24.8	n.a.	24.4	n.a.
RaRV1-MPa ^2^	n.a.	n.a.	n.a.	19.0

^1^ MPb of RaRV1 was compared to all MP, MPa, and MPb complete segments published; ^2^ Comparison of RaRV1-MPb to RaRV1-MPa only.

**Table 3:** Symptoms and other viruses detected in plants infected with the raspberry rubodvirus 1 (RaRV1). The original HTS-sequenced sample (V2) is shown on the first row; GPS indicates the location of the town, village, or field in which the sample was taken; Photo = letter of photo in Figure 5; * Young shoots were inspected only; ^1^ Source plant was transferred from RBIP Holovousy to Biology Centre CAS, Ceske Budejovice, a decade ago. Then, it was grown in a glasshouse of Biology Centre CAS.

Sample	Location	GPS	Cultivar	Other Viruses Detected	Symptoms	Photo
V2	Volanice	50.3351314N 15.3996269E	Canby	RBDV, RVCV	Vein clearing, mosaic, leaf curl	B
V4	Volanice	50.3351314N 15.3996269E	Canby	RBDV, RVCV	Vein clearing, mosaic, leaf curl	C
V5	Volanice	50.3351314N 15.3996269E	Canby	RBDV, RVCV	Vein clearing, yellowing	D
MC1	Míčov	49.9036772N 15.6046294E	Unknown	RaEV1, RVCV	Vein clearing, leaf curl, necrosis	E
MC2	Míčov	49.9036772N 15.6046294E	Unknown	RBDV, RVCV	Vein clearing, yellowing/reddening, necrosis	F
CH525	Chlum	50.3792903N 15.6024481E	Unknown	BRNV, RVCV	Vein clearing, mosaic	G
NB1	Nový Bydžov	50.2345183N 15.4883225E	Bulharský rubín	RaEV1, RBDV, RLBV, RVCV	Yellow blotches, leaf malformation	H
D23	Doudleby	50.1014906N, 16.2515422E	Canby	BRNV, RBDV, RLMV	No symptoms *	I
H1	Holovousy	Unknown ^1^	Malling Jewel 4	BRNV, RBDV, RLMV	Leaf curling	J
O4M	Ostroměř	50.3722167N, 15.5470811E	Unknown	RaEV1	Vein clearing, yellowing	K

**Table 4:** The occurrence and similarity of MPa and MPb fragments of RaRV1-positive isolates to isolate V2. Length—length of sequenced region (for isolate V2 it is the full length of particular ORF); nt/aa changes—changes in nucleotides/amino acids in comparison to V2 isolate.

Isolate	Locality	MPa				MPb			
		Detected	acc.no.	Length (nt)	nt/aa Changes	Detected	acc.no.	Length (nt)	nt/aa Changes
V2	Volanice	yes	PP732067	1315	0/0	yes	PP732068	1080	0/0
V4	Volanice	yes	PP934000	705	1/0	yes	PP942705	417	1/0
V5	Volanice	yes	PP934001	705	1/0	yes	PP934002	1054	3/2
MC1	Míčov	yes	PP934003	705	1/0	yes	PP934004	416	3/2
MC2	Míčov	yes	PP934005	688	1/0	yes	PP934006	419	2/1
O4M	Ostroměř	yes	PP977435 ^1^	688	0/0	yes	PP977434 ^2^	1038	4/3
NB1	Nový Bydžov	yes	PP942707	705	1/STOP ^3^	no ^2^	---	---	---
H1	Holovousy	yes	PP942706	705	1/0	no ^2^	---	---	---
CH525	Chlum	no ^1^	---	---	---	no ^2^	---	---	---
D23	Doudleby	no ^1^	---	---	---	no ^2^	---	---	---
*O. minutus*	Volanice	no ^1^	---	---	---	yes	PP933998	434	1/0
*A. idaei*	Volanice	no ^1^	---	---	---	yes	PP933999	422	1/0

^1^ confirmed with six different primer pairs; ^2^ confirmed with two different primer pairs; ^3^ premature STOP-codon after 1 nt insertion.

## Data Availability

The data presented in this study were deposited in the NCBI SRA storage repository under BioProjectID PRJNA1028176—experiment SRX24439621 and in the GenBank database under accession numbers PP732065-PP732070, PP933998-PP934006, PP942705-PP942707, and PP977434-PP977435. The data are also available upon request.

## Supplementary Materials

The following supporting information can be downloaded at: https://www.mdpi.com/article/10.3390/v16071074/s1, Table S1: Plant and arthropod samples analysed in this study; Table S2: Primers used in this study; Table S3: Similarities of selected plant phenuivirids and RaRV1.
