# Supplementary material for: Molecular Characterization of a Novel Rubodvirus Infecting Raspberries
Source: Viruses. 2024 Jul 3;16(7):1074. doi: 10.3390/v16071074 (PMC11281551; doi:10.3390/v16071074)
Supplement: Supplementary file 1 [file viruses-16-01074-s001.zip › Table S2.pdf]

<u>Table S2: Primers used in this study.</u> (A) primers for RaRV1 detection and sequencing; (B) primers for other viruses detection and for aphid plant diet detection. For UTR and RACE sequencing, different combinations with the other primers were used. References used in (B) are included also in the main text.

A)

**RdRP**			
name	*sequence (5' → 3´)*	*product size (bp)*	*purpose*
3164<br>3692	CTTGGGGATGTCTTTGTATTTGAT<br>CCATTTCGGCCTCAATCCCA	**1243**	internal region sequencing
3691<br>3690	AACTGACTCCGCTAAAGGCC<br>CGACTGGACCATAGACACAGG	**1357**	internal region sequencing
3689<br>3688	AGTTGCATGCAGCCTCTGAT<br>GCTGTTTCCAAAAGCAAGCC	**1265**	internal region sequencing
3131<br>3130	CTCTCGACTTGCCTCTTCTG<br>GGCATGCCAAATTCCTTCAG	**845**	internal region sequencing
3674<br>3673	ATGAGTGATCAATGCCTGCT<br>TGCAGTCCAACCAGAGCTTT	**1506**	internal region sequencing
3672<br>3671	GAAGCATCTCCACTCTTGGCT<br>ACACACGCAGGATTCTACATCA	**1364**	internal region sequencing
3762<br>3761	ACAAGTCTCTAAGGGTGCAAGG<br>ACTGCTAAAAAGCTTAATCTTGAG	**1164**	internal region sequencing
3670<br>3761	TGATGACCACTCAGTGCACA<br>ACTGCTAAAAAGCTTAATCTTGAG	**1116**	internal region sequencing
3179<br>3189	TGGGCTGGAAGAGATCAAAC<br>TCCAAAATTACAATTCGTGGAAATTAAAG	**589**	internal region sequencing
**3571**	ACACAAAGACCCCCTCCA	---	5'- RACE and 5'-end sequencing
**3165**	GAGGATGACGGTAGGCAAAT	---	5'- RACE and 5'-end sequencing
**3166**	CTTGGCACAGAAAGCAAACA	---	5'- RACE and 5'-end sequencing
**3167**	TCCAGAATCCAAAAGCCCTT	---	5'- RACE and 5'-end sequencing
**3760**	TGAGCAAGAAATGACTTCAGC	---	3'-RACE and 3'-end sequencing
**3669**	CACATGTGCAATGCAAGGGA	---	3'-RACE and 3'-end sequencing

<u>Position of RdRP-primers on RNA1 segment:</u>

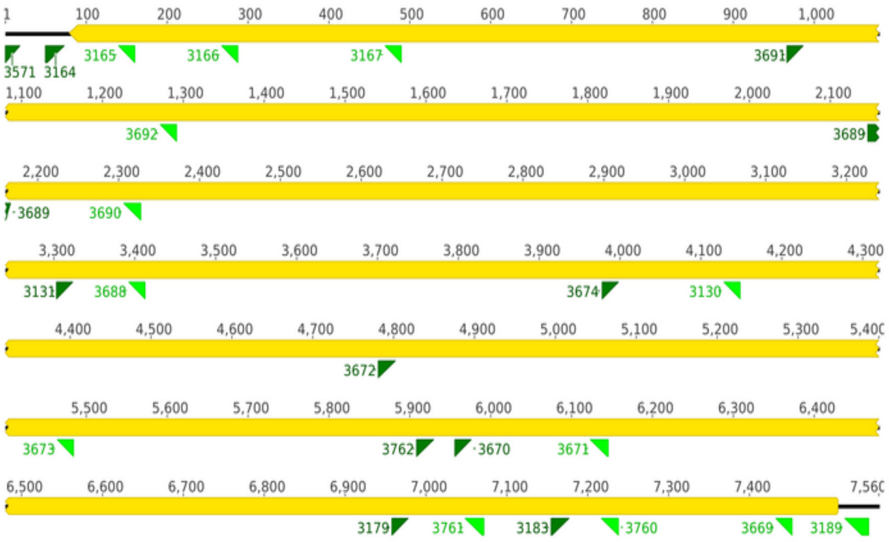

MPa			
name	*sequence (5’ → 3´)*	*product size (bp)*	*purpose*
3168	CATATTCTATGGAGAGTTTAAAATCAATCA		internal region sequencing
3124	AACATGTGGGTTCAGCATCA	1461	
3127	AAGCCTCCCTTGTCTGTTTC		internal region sequencing
3572	AATGGCGATGAACAGCGATA	774	
3180	AGGAAACCCTCTTCAGGACT		internal region sequencing
3572	AATGGCGATGAACAGCGATA	513	
3170	GTTTCTTTGCATGACTTTCGGA	---	5’- RACE and 5’-end sequencing
3171	AGTTGTCCTAACTGTTGGCA	---	5’- RACE and 5’-end sequencing
3172	GCCTTTTGGTGCAAGACAAA	---	5’- RACE and 5’-end sequencing
3184	CAGATTTCCCACCTGTGACA	---	3’-RACE and 3’-end sequencing
3180	AGGAAACCCTCTTCAGGACT	---	3’-RACE and 3’-end sequencing

Position of MPa-primers on RNA2 segment:

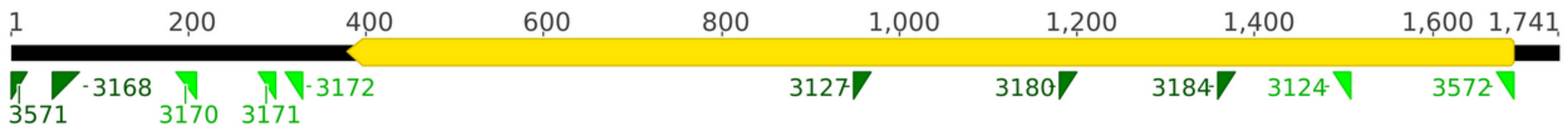

NP			
name	*sequence (5' → 3´)*	*product size (bp)*	*purpose*
3169	TGATGCTCACACAACCCATT	1220	internal region sequencing
3125	AGGCAATATCCGGCAAGATC		
3128	CTTCCCAACAGCAACTCTCA	842	internal region sequencing
3188	CAAGTTTCAGAATCTTCAAAGTTTACA		
**3128**	CTTCCCAACAGCAACTCTCA	553	internal region sequencing, **detection primers**
**3125**	AGGCAATATCCGGCAAGATC		
3571	ACACAAAGACCCCCTCCA	---	5'- RACE and 5'-end sequencing
3173	TGGCTGGCAATTTGGAATTC	---	5'- RACE and 5'-end sequencing
3174	CATGGTGAGCCCTTAGTTGT	---	5'- RACE and 5'-end sequencing
3177	TAGGTTTTGCCTCCGTCAAC	---	5'- RACE and 5'-end sequencing
3182	TCGAAGCTCAATGCCGTAAA	---	3'-RACE and 3'-end sequencing
3186	CACCATCTCTGTGAGTCCTG	---	3'-RACE and 3'-end sequencing
3758	AGCATCCACTCAACTCCTAGAG	---	3'-RACE and 3'-end sequencing
3759	TGGCAATTGATCTTAGCATCCAC	---	3'-RACE and 3'-end sequencing

Position of NP-primers on RNA3 segment:

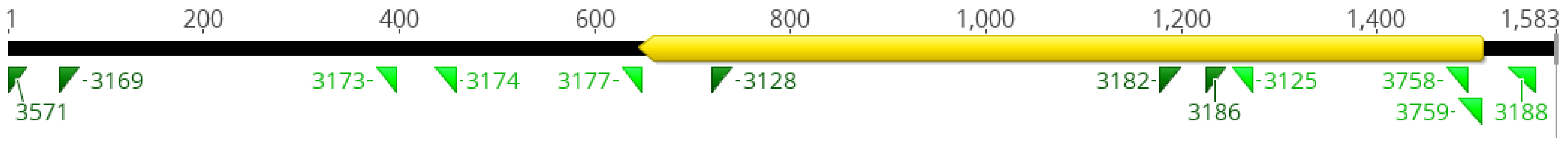

**MPb**			
name	*sequence (5' → 3´)*	*product size (bp)*	*purpose*
3129	CATCATCCAAGAACTCCCAGA	1097	internal region sequencing
3187	AAAGAACACACCCGTATAACCT		
3181	CGCCCAAGTGGTTATCAAGA	488	internal region sequencing
3187	AAAGAACACACCCGTATAACCT		
3175	TCAGATGGTTTCAAGAAGGTGA	---	5'- RACE and 5'-end sequencing
3176	ACAAGATCAAGACAAGAGGCAA	---	5'- RACE and 5'-end sequencing
3178	TGAGAGGGTCAATTGCACAA	---	5'- RACE and 5'-end sequencing
3181	CGCCCAAGTGGTTATCAAGA	---	3'-RACE and 3'-end sequencing

Position of MPb-primers on RNA4 segment:

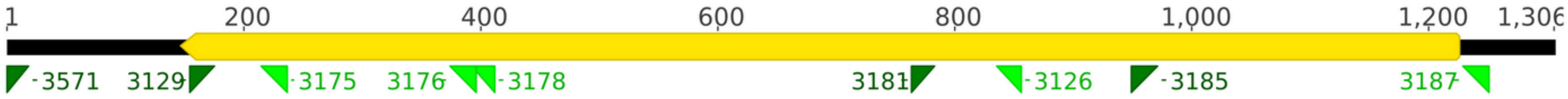

B)

Other viruses				
virus	*primer*	*sequence (5' → 3')*	*product size (bp)*	*citation*
BRNV	1153<br>1154	GCGCACTGAACCCAAGTTTA<br>CAACATCGAATCCCTCAAGC	502	James Hutton Ltd (https://www.hutton.ac.uk)
RLMV	CPhF<br>CPhR	CGAAACTTYTACGGGGAAC<br>CCTTTGAAYTCTTTAACATCGT	470	Tzanetakis et al., 2007
RVCV	F<br>R	CAGTAGAGGGGAGGCTCCTT<br>GTCCCACGTAAGGTTCTGGA	440	McGavin et al., 2011
RYNV	1752<br>1753	TCCAAAACCTCCCAGACCTAAAAC<br>ATAATCGCAAAAGGCAAGCCAC	350	Jones and McGavin, 2006
RLBV	F<br>R	ATCCAGTAGTGAACTCC<br>CACCATCAGGAACTTGTAATGTTT	560	McGavin et al., 2012
RBDV	CRF<br>CRR	TTTTCTACGGCTGCTGGTCT<br>GCATGTCCCTCAGTTTCGAT	370	James Hutton Ltd (https://www.hutton.ac.uk)
RpRSV	F1<br>R1	TGTGTCTGGTTTTGATGCT<br>GAGTGCGATAGGGGCTGTT	385	Ochoa-Corona et al., 2006
SLRSV	F<br>R	CCTCTCCAACCTGCTAGACT<br>AAGCGCATGAAGGTGTAACT	497	Postman et al., 2004
RaEV1	3280<br>3281	GTGAGCTAAGGCGAGCACA<br>AGGAGAAGGCGACGGACT	682	Koloniuk et al., 2023

Other primers				
primer	*sequence (5' → 3')*	*product size (bp)*	*purpose*	*citation*
AtropaNad2.1a<br>AtropaNad2.2b	GGACTCCTGACGTATACGAAGGATC<br>AGCAATGAGATTCCCCAATATCAT	188	aphid diet control	Thompson et al., 2003

References

Jones, A.; Mcgavin, W.; Geering, A.; Lockhart, B. Identification of Rubus yellow net virus as a distinct badnavirus and its detection by PCR in Rubus species and in aphids. *Ann. Appl. Biol.* **2006**, *141*, 1-10. DOI: 10.1111/j.1744-7348.2002.tb00189.x.

Koloniuk, I.; Fránová, J.; Přibylová, J.; Sarkisova, T.; Špak, J.; Tan, J. L.; Zemek, R.; Čmejla, R.; Rejlová, M.; Valentová, L.; Sedlák, J.; Holub, J.; Skalík, J.; Blystad, D. R.; Sapkota, B.; Hamborg, Z. Molecular Characterization of a Novel Enamovirus Infecting Raspberry. *Viruses* **2023**, *15(12)*, 2281. DOI: 10.3390/v15122281

McGavin, W.J.; Mitchell, C.; Cock, P.J.A.; Wright, K.M.; MacFarlane, S.A. Raspberry leaf blotch virus, a putative new member of the genus Emaravirus, encodes a novel genomic RNA. *J. Gen. Virol.* **2012**, *93(Pt 2)*, 430-437. DOI: 10.1099/vir.0.037937-0

Mcgavin, W.; Cock, P.; MacFarlane, S. Partial sequence and RT-PCR diagnostic test for the plant rhabdovirus Raspberry vein chlorosis virus. *Plant Pathol.* **2011**, *60*, 462-467. DOI: 10.1111/j.1365-3059.2010.02387.x.

Ochoa-Corona, F.M.; Lebas, B.S.M.; Tang, J.Z.; Bootten, T.J.; Stewart, F.J.; Harris, RElliott, D.R.; Alexander, B.J.R. RT-PCR detection and strain typing of Raspberry ringspot virus. Proceedings of the XXth International Symposium on Virus and Virus-Like Diseases of Temperate Fruit Crops & XIth International Symposium on Small Fruit Virus Diseases, Antalya, Turkey, 2006.

Postman, J.D.; Tzanetakis, I.E.; Martin, R.R. First Report of Strawberry latent ringspot virus in a Mentha sp. from North America. *Plant Dis.* **2004**, *88(8)*, 907. DOI: 10.1094/PDIS.2004.88.8.907B

Tzanetakis, I.E.; Halgren, A.; Mosier, N.; Martin, R.R. Identification and characterization of Raspberry mottle virus, a novel member of the Closteroviridae. *Virus Res.* **2007**, *127(1),* 26-33. DOI: 10.1016/j.virusres.2007.03.010
