# Supplementary material for: Molecular Characterization of a Novel Rubodvirus Infecting Raspberries
Source: Viruses. 2024 Jul 3;16(7):1074. doi: 10.3390/v16071074 (PMC11281551; doi:10.3390/v16071074)
Supplement: Supplementary file 1 [file viruses-16-01074-s001.zip › Table S3.pdf]

<u>Table S3: Similarities of selected plant phenuivirids and RaRV1.</u> All available reference sequences for phenuivirids were chosen (the same as in Fig. 4) are used, selected phlebovirus sequences were used as an outgroup (bold); A) aa identities of RdRPs, B) aa identities of NPs, C) aa identities of MPs, D) nt identities of RdRPs, E) nt identities of NPs, and F) nt identities of MPs.

A) RdRP (aa-identities)

	RUBOD_Qingdao RNA virus 3_LC726787	RUBOD_Apple rubbery wood virus 2_NC_055534	RUBOD_Grapevine Garan dmak virus_NC_078405	RUBOD_Apple rubbery wood virus 1_NC_055390	RUBOD_Grapevine Muscat rose virus_NC_078401	**RUBOD Raspberry rubodvirus 1**	COGU_Watermelon crinkle leaf-associated virus 1_NC_079048	COGU_Citrus virus A_NC_078103	COGU_Citrus concave gum-associated virus_NC_035759	COGU_Watermelon crinkle leaf-associated virus 2_NC_079050	COGU_Brassica campestris chinensis coguvirus 1_NC_079045	COGU_Grapevine associated cogu-like virus 1_NC_0784331	LAULA_Grapevine associated cogu-like virus 4_NC_078448	LAULA_Grapevine associated cogu-like virus 3_NC_0784401	LAULA_Grapevine associated cogu-like virus 2_NC_0784371	TENUI_European wheat striate mosaic virus_NC_0784091	TENUI_Tenuivirus oryzalbae_NC_036597	TENUI_Tenuivirus oryzaclavatae_NC_003755	TENUI_Tenuivirus oryzabrevis_NC_002323	MECHLORO_Melon chlorotic spot virus_NC_040450	**OUT_Phlebovirus_NC_078067**
RUBOD_Qingdao RNA virus 3_LC726787		25.3	24.1	26.3	22.9	25.3	17.0	17.0	16.6	17.3	17.1	16.4	16.0	15.5	14.4	11.5	11.5	11.5	11.6	11.7	14.5
RUBOD_Apple rubbery wood virus 2_NC_055534	25.3		60.6	59.4	50.9	33.7	17.4	16.8	16.7	16.8	15.9	16.6	16.0	15.6	15.3	11.3	11.1	11.3	11.7	11.5	13.8
RUBOD_Grapevine Garan dmak virus_NC_078405	24.1	60.6		52.2	52.5	30.4	16.4	15.1	15.2	15.9	14.8	14.5	14.3	14.5	14.9	12.2	11.7	11.9	12.5	12.4	12.4
RUBOD_Apple rubbery wood virus 1_NC_055390	26.3	59.4	52.2		49.6	34.1	17.3	17.2	16.9	16.6	16.2	16.2	15.7	15.0	15.2	11.8	11.4	11.5	11.7	11.7	15.2
RUBOD_Grapevine Muscat rose virus_NC_078401	22.9	50.9	52.5	49.6		30.4	16.9	15.5	15.2	16.8	15.0	14.9	15.2	15.5	15.9	12.5	11.9	12.4	12.9	12.5	13.7
**RUBOD Raspberry rubodvirus 1**	25.3	33.7	30.4	34.1	30.4		16.5	16.1	16.4	16.4	16.0	15.8	15.8	15.5	15.0	11.2	11.3	11.0	11.8	11.3	13.8
COGU_Watermelon crinkle leaf-associated virus 1_NC_079048	17.0	17.4	16.4	17.3	16.9	16.5		63.0	62.5	58.8	54.7	38.1	30.5	30.7	30.6	15.4	15.0	15.2	15.6	16.1	18.0
COGU_Citrus virus A_NC_078103	17.0	16.8	15.1	17.2	15.5	16.1	63.0		76.6	57.4	55.9	38.4	30.9	28.9	28.5	12.9	12.6	12.9	12.8	12.9	17.1
COGU_Citrus concave gum-associated virus_NC_035759	16.6	16.7	15.2	16.9	15.2	16.4	62.5	76.6		57.9	54.9	37.2	31.4	28.7	28.6	13.1	12.7	12.9	13.2	13.7	17.7
COGU_Watermelon crinkle leaf-associated virus 2_NC_079050	17.3	16.8	15.9	16.6	16.8	16.4	58.8	57.4	57.9		52.0	38.1	29.7	29.8	29.5	15.1	15.2	15.2	15.1	15.7	17.8
COGU_Brassica campestris chinensis coguvirus 1_NC_079045	17.1	15.9	14.8	16.2	15.0	16.0	54.7	55.9	54.9	52.0		37.8	29.9	28.3	28.1	12.9	12.8	13.2	13.1	13.5	17.5
COGU_Grapevine associated cogu-like virus 1_NC_0784331	16.4	16.6	14.5	16.2	14.9	15.8	38.1	38.4	37.2	38.1	37.8		28.4	26.8	26.6	12.4	12.5	12.6	12.7	13.4	16.7
LAULA_Grapevine associated cogu-like virus 4_NC_078448	16.0	16.0	14.3	15.7	15.2	15.8	30.5	30.9	31.4	29.7	29.9	28.4		60.7	60.0	12.4	12.5	13.0	12.2	12.9	16.9
LAULA_Grapevine associated cogu-like virus 3_NC_0784401	15.5	15.6	14.5	15.0	15.5	15.5	30.7	28.9	28.7	29.8	28.3	26.8	60.7		75.9	12.8	13.4	13.5	13.6	13.4	15.7
LAULA_Grapevine associated cogu-like virus 2_NC_0784371	14.4	15.3	14.9	15.2	15.9	15.0	30.6	28.5	28.6	29.5	28.1	26.6	60.0	75.9		12.9	13.2	13.4	13.6	13.6	15.3
TENUI_European wheat striate mosaic virus_NC_0784091	11.5	11.3	12.2	11.8	12.5	11.2	15.4	12.9	13.1	15.1	12.9	12.4	12.4	12.8	12.9		45.3	44.8	32.1	25.4	18.1
TENUI_Tenuivirus oryzalbae_NC_036597	11.5	11.1	11.7	11.4	11.9	11.3	15.0	12.6	12.7	15.2	12.8	12.5	12.5	13.4	13.2	45.3		58.1	30.6	25.0	18.2
TENUI_Tenuivirus oryzaclavatae_NC_003755	11.5	11.3	11.9	11.5	12.4	11.0	15.2	12.9	12.9	15.2	13.2	12.6	13.0	13.5	13.4	44.8	58.1		30.8	26.1	18.2
TENUI_Tenuivirus oryzabrevis_NC_002323	11.6	11.7	12.5	11.7	12.9	11.8	15.6	12.8	13.2	15.1	13.1	12.7	12.2	13.6	13.6	32.1	30.6	30.8		25.4	17.9
MECHLORO_Melon chlorotic spot virus_NC_040450	11.7	11.5	12.4	11.7	12.5	11.3	16.1	12.9	13.7	15.7	13.5	13.4	12.9	13.4	13.6	25.4	25.0	26.1	25.4		17.3
**OUT_Phlebovirus_NC_078067**	14.5	13.8	12.4	15.2	13.7	13.8	18.0	17.1	17.7	17.8	17.5	16.7	16.9	15.7	15.3	18.1	18.2	18.2	17.9	17.3	

B) NP (aa-identities)

	RUBOD_Qingdao RNA virus 3_LC726788	Rubod_Raspberry rubodvirus 1	RUBOD_Apple rubbery wood virus 1_NC_055392	RUBOD_Apple rubbery wood virus 2_NC_055533	RUBOD_Apple rubbery wood virus 2_NC_055535	RUBOD_Grapevine Garan dmak virus_NC_078404	RUBOD_Grapevine Muscat rose virus_NC_078402	COGU_Brassica campestris chinensis coguvirus 1_NC_079046	COGU_Citrus concave gum-associated virus_NC_035454	COGU_Citrus virus A_NC_0781042gp2	COGU_Watermelon crinkle leaf-associated virus 1_NC_079047	COGU_Watermelon crinkle leaf-associated virus 2_NC_079049	LAULA_Grapevine associated cogu-like virus 4_NC_078446	COGU_Grapevine associated cogu-like virus 1_NC_0784323	LAULA_Grapevine associated cogu-like virus 2_NC_0784363	LAULA_Grapevine associated cogu-like virus 3_NC_0784393	LAULA_Laurel Lake virus_NC_043681	**OUT_Phlebovirus_NC_078066**	TENUI_European wheat striate mosaic virus_NC_0784073	TENUI_Tenuivirus echinochloae_NC_038934	TENUI_Tenuivirus oryzalbae_NC_036602	TENUI_Tenuivirus urochloae_NC_038757	TENUI_Tenuivirus persotritici_NC_038750	TENUI_Tenuivirus zeae_NC_038754	TENUI_Tenuivirus oryzabrevis_NC_002327	MECHLORO_Melon chlorotic spot virus_NC_040454
RUBOD_Qingdao RNA virus 3_LC726788		23.8	23.8	21.0	21.6	21.6	21.5	10.8	16.4	16.8	15.6	18.3	17.0	17.2	14.0	14.0	13.5	12.1	8.4	12.6	11.8	12.4	11.2	9.8	9.8	11.0
**Rubod_Raspberry rubodvirus 1**	23.8		42.6	44.9	41.5	41.2	42.1	11.9	17.3	16.4	17.1	16.1	20.0	15.3	15.3	16.4	18.0	18.8	11.7	11.5	10.7	10.7	11.6	9.8	15.1	13.6
RUBOD_Apple rubbery wood virus 1_NC_055392	23.8	42.6		67.8	68.4	67.7	68.0	10.1	16.7	17.4	16.0	15.7	20.8	15.6	16.8	18.9	18.1	21.3	10.8	12.7	13.1	12.8	13.8	11.9	14.2	14.1
RUBOD_Apple rubbery wood virus 2_NC_055533	21.0	44.9	67.8		77.4	75.3	73.9	10.6	18.1	17.4	17.4	16.9	20.8	15.3	17.2	17.9	18.8	18.0	12.0	14.2	14.3	14.0	14.1	12.5	14.5	13.5
RUBOD_Apple rubbery wood virus 2_NC_055535	21.6	41.5	68.4	77.4		89.6	73.4	9.7	16.7	16.8	17.4	16.3	20.1	15.0	18.6	18.9	18.4	18.0	13.5	15.2	14.0	14.6	15.9	13.9	13.6	13.1
RUBOD_Grapevine Garan dmak virus_NC_078404	21.6	41.2	67.7	75.3	89.6		73.8	9.9	17.8	17.1	17.6	15.4	20.5	15.0	17.9	18.2	18.4	17.6	12.0	14.5	13.7	14.9	14.7	14.2	14.5	13.1
RUBOD_Grapevine Muscat rose virus_NC_078402	21.5	42.1	68.0	73.9	73.4	73.8		9.9	18.9	17.1	17.1	17.4	19.8	15.0	18.2	18.2	20.5	19.5	11.2	13.7	13.2	13.2	13.6	13.1	13.7	14.2
COGU_Brassica campestris chinensis coguvirus 1_NC_079046	10.8	11.9	10.1	10.6	9.7	9.9	9.9		25.4	26.5	27.7	22.6	16.0	15.8	10.9	10.9	11.1	9.1	5.7	14.6	12.4	13.2	13.1	7.2	8.3	7.6
COGU_Citrus concave gum-associated virus_NC_035454	16.4	17.3	16.7	18.1	16.7	17.8	18.9	25.4		59.0	50.7	45.0	25.1	28.8	16.3	16.9	15.9	11.2	8.6	10.8	10.3	11.6	12.6	10.5	10.9	11.5
COGU_Citrus virus A_NC_0781042gp2	16.8	16.4	17.4	17.4	16.8	17.1	17.1	26.5	59.0		47.3	39.8	23.2	27.2	16.8	17.3	15.5	9.7	9.6	12.1	11.5	11.5	12.2	10.0	9.7	11.5
COGU_Watermelon crinkle leaf-associated virus 1_NC_079047	15.6	17.1	16.0	17.4	17.4	17.6	17.1	27.7	50.7	47.3		47.7	25.7	30.6	19.0	18.8	18.5	11.5	9.2	13.1	12.8	13.1	12.7	10.1	11.8	12.9
COGU_Watermelon crinkle leaf-associated virus 2_NC_079049	18.3	16.1	15.7	16.9	16.3	15.4	17.4	22.6	45.0	39.8	47.7		23.9	29.3	17.7	16.5	16.6	11.0	8.4	13.4	12.8	12.2	12.3	8.8	11.1	11.7
LAULA_Grapevine associated cogu-like virus 4_NC_078446	17.0	20.0	20.8	20.8	20.1	20.5	19.8	16.0	25.1	23.2	25.7	23.9		21.6	26.4	27.1	25.6	17.4	12.9	15.7	14.0	15.0	16.0	13.4	13.7	14.6
COGU_Grapevine associated cogu-like virus 1_NC_0784323	17.2	15.3	15.6	15.3	15.0	15.0	15.0	15.8	28.8	27.2	30.6	29.3	21.6		16.4	15.8	17.0	13.1	8.7	11.5	11.3	11.6	12.7	10.4	9.8	10.6
LAULA_Grapevine associated cogu-like virus 2_NC_0784363	14.0	15.3	16.8	17.2	18.6	17.9	18.2	10.9	16.3	16.8	19.0	17.7	26.4	16.4		74.7	68.5	18.1	13.1	13.6	12.3	12.9	12.7	13.3	12.4	16.0
LAULA_Grapevine associated cogu-like virus 3_NC_0784393	14.0	16.4	18.9	17.9	18.9	18.2	18.2	10.9	16.9	17.3	18.8	16.5	27.1	15.8	74.7		70.1	17.4	12.7	11.1	10.5	10.5	12.1	11.4	9.4	13.8
LAULA_Laurel Lake virus_NC_043681	13.5	18.0	18.1	18.8	18.4	18.4	20.5	11.1	15.9	15.5	18.5	16.6	25.6	17.0	68.5	70.1		15.8	11.9	10.3	9.3	9.9	10.0	12.5	11.6	13.6
**OUT_Phlebovirus_NC_078066**	12.1	18.8	21.3	18.0	18.0	17.6	19.5	9.1	11.2	9.7	11.5	11.0	17.4	13.1	18.1	17.4	15.8		16.3	12.9	13.1	13.5	13.6	15.9	13.1	15.7
TENUI_European wheat striate mosaic virus_NC_0784073	8.4	11.7	10.8	12.0	13.5	12.0	11.2	5.7	8.6	9.6	9.2	8.4	12.9	8.7	13.1	12.7	11.9	16.3		32.2	31.3	32.0	30.6	35.0	16.6	13.5
TENUI_Tenuivirus echinochloae_NC_038934	12.6	11.5	12.7	14.2	15.2	14.5	13.7	14.6	10.8	12.1	13.1	13.4	15.7	11.5	13.6	11.1	10.3	12.9	32.2		89.6	89.6	69.4	46.8	18.5	15.7
TENUI_Tenuivirus oryzalbae_NC_036602	11.8	10.7	13.1	14.3	14.0	13.7	13.2	12.4	10.3	11.5	12.8	12.8	14.0	11.3	12.3	10.5	9.3	13.1	31.3	89.6		92.8	69.9	47.5	17.6	15.0
TENUI_Tenuivirus urochloae_NC_038757	12.4	10.7	12.8	14.0	14.6	14.9	13.2	13.2	11.6	11.5	13.1	12.2	15.0	11.6	12.9	10.5	9.9	13.5	32.0	89.6	92.8		70.5	48.1	17.6	15.9
TENUI_Tenuivirus persotritici_NC_038750	11.2	11.6	13.8	14.1	15.9	14.7	13.6	13.1	12.6	12.2	12.7	12.3	16.0	12.7	12.7	12.1	10.0	13.6	30.6	69.4	69.9	70.5		43.5	19.0	14.5
TENUI_Tenuivirus zeae_NC_038754	9.8	9.8	11.9	12.5	13.9	14.2	13.1	7.2	10.5	10.0	10.1	8.8	13.4	10.4	13.3	11.4	12.5	15.9	35.0	46.8	47.5	48.1	43.5		17.2	15.0
TENUI_Tenuivirus oryzabrevis_NC_002327	9.8	15.1	14.2	14.5	13.6	14.5	13.7	8.3	10.9	9.7	11.8	11.1	13.7	9.8	12.4	9.4	11.6	13.1	16.6	18.5	17.6	17.6	19.0	17.2		13.9
MECHLORO_Melon chlorotic spot virus_NC_040454	11.0	13.6	14.1	13.5	13.1	13.1	14.2	7.6	11.5	11.5	12.9	11.7	14.6	10.6	16.0	13.8	13.6	15.7	13.5	15.7	15.0	15.9	14.5	15.0	13.9	

C) MP (aa identities)

	TENUI_Tenuivirus oryzalbae_NC_0365994	TENUI_Tenuivirus echinochloae_NC_03893544	TENUI_Tenuivirus urochloae_NC_038758	**Rubod_Raspberry rubodvirus 1 – MPa**	**Rubod_Raspberry rubodvirus 1 – MPb**	RUBOD__Grapevine Muscat rose virus_NC_078403	RUBOD_Apple rubbery wood virus 1_NC_055391	RUBOD__Apple rubbery wood virus 2_NC_055537	RUBOD__Grapevine Garan dmak virus_NC_078406	RUBOD__Apple rubbery wood virus 2_NC_055536	Phlebo_OUT_OR610328	COGU_Brassica campestris chinensis coguvirus 1_NC_079046	COGU_Watermelon crinkle leaf-associated virus 2_NC_079049	COGU_Watermelon crinkle leaf-associated virus 1_NC_079047	COGU_Citrus concave gum-associated virus_NC_03545446	COGU_Citrus virus A_NC_0781042gp1	COGU_Grapevine associated cogu-like virus 1_NC_0784342	LAULA_Grapevine associated cogu-like virus 4_NC_078447	LAULA_Grapevine associated cogu-like virus 3_NC_0784382	LAULA_Grapevine associated cogu-like virus 2_NC_0784352
TENUI_Tenuivirus oryzalbae_NC_0365994		88.7	90.5	7.6	8.4	8.6	7.3	8.2	7.7	9.5	7.2	9.9	8.6	8.8	8.0	9.1	8.6	7.0	6.2	5.9
TENUI_Tenuivirus echinochloae_NC_03893544	88.7		93.3	7.3	8.1	8.9	7.3	8.2	8.0	9.5	7.8	8.8	8.6	8.8	8.0	9.1	8.9	7.5	6.2	5.9
TENUI_Tenuivirus urochloae_NC_038758	90.5	93.3		7.0	7.8	8.0	7.0	8.5	7.4	8.9	8.3	9.1	8.3	8.8	8.0	9.7	8.9	7.0	6.2	5.9
**Rubod_Raspberry rubodvirus 1 – MPb**	7.6	7.3	7.0		15.2	16.1	18.0	17.1	17.6	17.8	8.1	9.5	10.8	11.1	10.3	10.0	7.2	9.2	8.8	9.0
**Rubod_Raspberry rubodvirus 1 – MPa**	8.4	8.1	7.8	15.2		32.9	35.7	34.3	34.5	35.5	10.3	7.8	8.8	10.4	8.9	7.8	9.7	9.3	9.7	9.7
RUBOD__Grapevine Muscat rose virus_NC_078403	8.6	8.9	8.0	16.1	32.9		53.7	56.4	55.0	60.0	10.1	10.8	9.9	11.2	11.1	10.9	9.4	8.2	8.7	8.5
RUBOD_Apple rubbery wood virus 1_NC_055391	7.3	7.3	7.0	18.0	35.7	53.7		58.1	59.8	60.5	7.5	9.5	9.8	9.6	9.0	10.5	9.3	9.0	8.6	8.4
RUBOD__Apple rubbery wood virus 2_NC_055537	8.2	8.2	8.5	17.1	34.3	56.4	58.1		64.0	66.3	9.1	9.5	10.3	12.0	10.7	10.7	8.8	9.8	10.1	10.1
RUBOD__Grapevine Garan dmak virus_NC_078406	7.7	8.0	7.4	17.6	34.5	55.0	59.8	64.0		77.9	9.4	9.6	10.8	11.9	11.2	10.8	8.9	9.0	10.2	9.9
RUBOD__Apple rubbery wood virus 2_NC_055536	9.5	9.5	8.9	17.8	35.5	60.0	60.5	66.3	77.9		10.7	9.3	9.9	11.1	10.5	9.3	8.9	9.9	9.7	9.5
**Phlebo_OUT_OR610328**	7.2	7.8	8.3	8.1	10.3	10.1	7.5	9.1	9.4	10.7		12.8	16.3	13.6	13.1	12.6	13.3	12.0	12.9	12.9
COGU_Brassica campestris chinensis coguvirus 1_NC_079046	9.9	8.8	9.1	9.5	7.8	10.8	9.5	9.5	9.6	9.3	12.8		33.1	37.9	36.9	38.7	19.2	22.7	22.0	22.0
COGU_Watermelon crinkle leaf-associated virus 2_NC_079049	8.6	8.6	8.3	10.8	8.8	9.9	9.8	10.3	10.8	9.9	16.3	33.1		45.1	43.7	44.4	21.7	23.1	23.0	23.2
COGU_Watermelon crinkle leaf-associated virus 1_NC_079047	8.8	8.8	8.8	11.1	10.4	11.2	9.6	12.0	11.9	11.1	13.6	37.9	45.1		50.3	49.5	20.9	24.1	24.4	24.7
COGU_Citrus concave gum-associated virus_NC_03545446	8.0	8.0	8.0	10.3	8.9	11.1	9.0	10.7	11.2	10.5	13.1	36.9	43.7	50.3		64.5	20.9	22.2	22.4	22.4
COGU_Citrus virus A_NC_0781042gp1	9.1	9.1	9.7	10.0	7.8	10.9	10.5	10.7	10.8	9.3	12.6	38.7	44.4	49.5	64.5		24.7	21.4	23.9	23.9
COGU_Grapevine associated cogu-like virus 1_NC_0784342	8.6	8.9	8.9	7.2	9.7	9.4	9.3	8.8	8.9	8.9	13.3	19.2	21.7	20.9	20.9	24.7		25.7	27.6	27.8
LAULA_Grapevine associated cogu-like virus 4_NC_078447	7.0	7.5	7.0	9.2	9.3	8.2	9.0	9.8	9.0	9.9	12.0	22.7	23.1	24.1	22.2	21.4	25.7		51.3	50.9
LAULA_Grapevine associated cogu-like virus 3_NC_0784382	6.2	6.2	6.2	8.8	9.7	8.7	8.6	10.1	10.2	9.7	12.9	22.0	23.0	24.4	22.4	23.9	27.6	51.3		94.3
LAULA_Grapevine associated cogu-like virus 2_NC_0784352	5.9	5.9	5.9	9.0	9.7	8.5	8.4	10.1	9.9	9.5	12.9	22.0	23.2	24.7	22.4	23.9	27.8	50.9	94.3	

D) RdRP (nt identities)

	TENUI_Tenuivirus oryzalbae_NC_036597	TENUI_European wheat striate mosaic virus_NC_078409	TENUI_Tenuivirus oryzaclavatae_NC_003755 (reversed)	OUT_Phlebovirus_NC_078067	LAULA_Grapevine associated cogu-like virus 4_NC_078448	LAULA_Grapevine associated cogu-like virus 2_NC_078437	LAULA_Grapevine associated cogu-like virus 3_NC_078440	COGU_Grapevine associated cogu-like virus 1_NC_078433	TENUI_Tenuivirus oryzabrevis_NC_002323	MECHLORO_Melon chlorotic spot virus_NC_040450 (reversed)	COGU_Brassica campestris chinensis coguvirus 1_NC_079045	COGU_Watermelon crinkle leaf-associated virus 2_NC_079050	COGU_Watermelon crinkle leaf-associated virus 1_NC_079048	COGU_Citrus concave gum-associated virus_NC_035759	COGU_Citrus virus A_NC_078103	RUBOD_Grapevine Muscat rose virus_NC_078401	RUBOD_Apple rubbery wood virus 1_NC_055390	RUBOD_Apple rubbery wood virus 2_NC_055534	RUBOD_Grapevine Garan dmak virus_NC_078405	RUBOD_Qingdao_RNA_Virus_3_LC726787	RUBOD_Raspberry rubodvirus 1
TENUI_Tenuivirus oryzalbae_NC_036597		44.4	47.7	25.3	27	28	27.6	26.4	35.6	31.6	27.4	27.8	27.7	28.2	28.2	27.3	26.4	25.9	27.5	26.6	26
TENUI_European wheat striate mosaic virus_NC_078409	44.4		43.3	26.1	27.7	28.9	28.6	26.7	37.4	33.8	27.3	28.2	28.6	27.9	28.5	28.1	26.6	25.8	28	27.7	26.1
TENUI_Tenuivirus oryzaclavatae_NC_003755 (reversed)	47.7	43.3		25.8	26.7	27.5	27.8	25.8	35.6	32.5	27.8	28.3	27.6	28.6	29	27.8	26.5	25.5	27.6	26.9	25.6
OUT_Phlebovirus_NC_078067	25.3	26.1	25.8		29.9	27.8	28.5	29.9	26.2	25	30.8	31.5	31.7	32	32	25.7	27.5	27.8	25.3	28	27.2
LAULA_Grapevine associated cogu-like virus 4_NC_078448	27	27.7	26.7	29.9		52.7	53.6	38	26.6	26	39.9	40.2	40.3	40.1	40.5	28.9	31	31	28.2	32.2	31.2
LAULA_Grapevine associated cogu-like virus 2_NC_078437	28	28.9	27.5	27.8	52.7		61.9	36.2	28.1	28.2	38.4	38.4	39.1	38.5	38.7	31.1	29.8	29.7	30.2	30.8	29.3
LAULA_Grapevine associated cogu-like virus 3_NC_078440	27.6	28.6	27.8	28.5	53.6	61.9		36.1	27.5	27.8	37.9	38	38.1	37.9	39.1	31.2	29.7	29.9	30.3	30.7	29.9
COGU_Grapevine associated cogu-like virus 1_NC_078433	26.4	26.7	25.8	29.9	38	36.2	36.1		27.4	25.6	43.1	44.6	43.7	43.3	44	29	30.9	30.9	27.8	31.3	31.8
TENUI_Tenuivirus oryzabrevis_NC_002323	35.6	37.4	35.6	26.2	26.6	28.1	27.5	27.4		33.8	28.4	28.4	28	27.9	28.3	28.6	26.2	26.2	28.2	26.4	25.8
MECHLORO_Melon chlorotic spot virus_NC_040450 (reversed)	31.6	33.8	32.5	25	26	28.2	27.8	25.6	33.8		27.7	28.2	28.5	28	28.4	27.9	25.7	26	27.4	25.2	24.1
COGU_Brassica campestris chinensis coguvirus 1_NC_079045	27.4	27.3	27.8	30.8	39.9	38.4	37.9	43.1	28.4	27.7		53.6	54.3	54.5	55	30.2	32.3	31.8	28.7	33	31.9
COGU_Watermelon crinkle leaf-associated virus 2_NC_079050	27.8	28.2	28.3	31.5	40.2	38.4	38	44.6	28.4	28.2	53.6		58	57.7	58.3	30	33.2	31.9	29	34	32.5
COGU_Watermelon crinkle leaf-associated virus 1_NC_079048	27.7	28.6	27.6	31.7	40.3	39.1	38.1	43.7	28	28.5	54.3	58		59.7	60.2	30.9	31.8	32.1	28.6	33.5	31.9
COGU_Citrus concave gum-associated virus_NC_035759	28.2	27.9	28.6	32	40.1	38.5	37.9	43.3	27.9	28	54.5	57.7	59.7		66.5	29.8	31.7	31.6	28.5	33	32.6
COGU_Citrus virus A_NC_078103	28.2	28.5	29	32	40.5	38.7	39.1	44	28.3	28.4	55	58.3	60.2	66.5		30.6	32.6	32	29.3	33.6	31.4
RUBOD_Grapevine Muscat rose virus_NC_078401	27.3	28.1	27.8	25.7	28.9	31.1	31.2	29	28.6	27.9	30.2	30	30.9	29.8	30.6		49	49.5	51.5	34.2	35.9
RUBOD_Apple rubbery wood virus 1_NC_055390	26.4	26.6	26.5	27.5	31	29.8	29.7	30.9	26.2	25.7	32.3	33.2	31.8	31.7	32.6	49		55.8	49.4	37.3	38.6
RUBOD_Apple rubbery wood virus 2_NC_055534	25.9	25.8	25.5	27.8	31	29.7	29.9	30.9	26.2	26	31.8	31.9	32.1	31.6	32	49.5	55.8		54.2	36.5	38.3
RUBOD_Grapevine Garan dmak virus_NC_078405	27.5	28	27.6	25.3	28.2	30.2	30.3	27.8	28.2	27.4	28.7	29	28.6	28.5	29.3	51.5	49.4	54.2		33.8	35.3
RUBOD_Qingdao_RNA_Virus_3_LC726787	26.6	27.7	26.9	28	32.2	30.8	30.7	31.3	26.4	25.2	33	34	33.5	33	33.6	34.2	37.3	36.5	33.8		35.7
**RUBOD_Raspberry rubodvirus 1**	26	26.1	25.6	27.2	31.2	29.3	29.9	31.8	25.8	24.1	31.9	32.5	31.9	32.6	31.4	35.9	38.6	38.3	35.3	35.7	

E) NP (nt identities)

	TENUI_Tenuivirus oryzabrevis_NC_002327	LAULA_Grapevine associated cogu-like virus 4_NC_078446	TENUI_Tenuivirus persotritici_NC_038750	TENUI_Tenuivirus oryzalbae_NC_036602	TENUI_Tenuivirus echinochloae_NC_038934	TENUI_Tenuivirus urochloae_NC_038757	PHLEBO OUT LC726788	COGU_Grapevine associated cogu-like virus 1_NC_078432	**RUBOD_Raspberry rubodvirus 1**	RUBOD_Grapevine Muscat rose virus_NC_078402	RUBOD_Apple rubbery wood virus 1_NC_055392	RUBOD_Grapevine Garan dmak virus_NC_078404	RUBOD_Apple rubbery wood virus 2_NC_055535	RUBOD_Apple rubbery wood virus 2_NC_055533	TENUI_European wheat striate mosaic virus_NC_078407	COGU_Watermelon crinkle leaf-associated virus 2_NC_079049	MECHLORO_Melon chlorotic spot virus_NC_040454	LAULA_Grapevine associated cogu-like virus 3_NC_078439	LAULA_Laurel Lake virus_NC_043681	LAULA_Grapevine associated cogu-like virus 2_NC_078436	COGU_Brassica campestris chinensis coguvirus 1_NC_079046	COGU_Watermelon crinkle leaf-associated virus 1_NC_079047	COGU_Citrus concave gum-associated virus_NC_035454	COGU_Citrus virus A_NC_078104
TENUI_Tenuivirus oryzabrevis_NC_002327		27.8	33.8	33.1	34.7	35.1	27.6	22.7	29.8	29.7	30.2	30.7	30.8	32.7	33	25.9	27.1	28.5	28.1	28.3	16.4	25.1	25.3	24.8
LAULA_Grapevine associated cogu-like virus 4_NC_078446	27.8		30	30.3	31.2	31.5	27.9	27.3	32	33.2	31.3	32.6	33	33.4	28.7	30.4	26.2	34.5	33.2	32	20.8	29.7	31.8	30.7
TENUI_Tenuivirus persotritici_NC_038750	33.8	30		66.3	65.7	66.7	27.5	24.8	31.1	28.3	28.4	30.9	30.6	31.7	39	23.7	26.4	28.2	28.2	29.2	17.2	25.3	25	24
TENUI_Tenuivirus oryzalbae_NC_036602	33.1	30.3	66.3		81.6	82.2	27.4	25.7	30	30.8	29.6	31.6	31.7	32.1	40.6	23.7	28.9	29	29.1	27.7	18.2	25.8	24.6	23.2
TENUI_Tenuivirus echinochloae_NC_038934	34.7	31.2	65.7	81.6		82.5	29.1	26	31.4	31.2	30.8	32.2	32.3	31.9	40.7	23.9	28.9	29	30.1	28.6	18.8	26.5	24.8	24.1
TENUI_Tenuivirus urochloae_NC_038757	35.1	31.5	66.7	82.2	82.5		29	26.9	32.2	31.8	30.7	32.5	31.7	33.1	40.1	23	27.7	28.5	29.1	28.7	18.5	26.2	24.6	24.2
PHLEBO OUT LC726788	27.6	27.9	27.5	27.4	29.1	29		28.4	33.4	32.2	33.2	32.4	32.6	33.3	25.7	29.1	26.8	27.2	25.7	26.5	21	31	30.5	29.2
COGU_Grapevine associated cogu-like virus 1_NC_078432	22.7	27.3	24.8	25.7	26	26.9	28.4		28.7	30.5	29.3	29.9	29.8	29.6	23.4	38.6	21.9	27	26.8	28.2	25.1	36.5	39.2	37.1
**RUBOD_Raspberry rubodvirus 1**	29.8	32	31.1	30	31.4	32.2	33.4	28.7		43.4	45.3	44.2	43.8	45.4	30.1	26.4	28.5	33.2	33.4	34.2	19.4	29.6	27.4	27.2
RUBOD_Grapevine Muscat rose virus_NC_078402	29.7	33.2	28.3	30.8	31.2	31.8	32.2	30.5	43.4		64.6	66.5	64.8	69.4	28.7	29.1	29.2	35	34.2	35.2	18.6	29.3	29.6	28.7
RUBOD_Apple rubbery wood virus 1_NC_055392	30.2	31.3	28.4	29.6	30.8	30.7	33.2	29.3	45.3	64.6		65.9	66.1	66.3	30	26.8	29.9	34.6	35	33.3	19.1	30.1	28.7	28.3
RUBOD_Grapevine Garan dmak virus_NC_078404	30.7	32.6	30.9	31.6	32.2	32.5	32.4	29.9	44.2	66.5	65.9		85.4	69.6	29.7	27.5	29	34.8	34.7	33.9	18.4	28.8	29.8	27
RUBOD_Apple rubbery wood virus 2_NC_055535	30.8	33	30.6	31.7	32.3	31.7	32.6	29.8	43.8	64.8	66.1	85.4		69.3	29.7	27.9	29.2	35	34.7	33.9	18.7	30.3	30	28
RUBOD_Apple rubbery wood virus 2_NC_055533	32.7	33.4	31.7	32.1	31.9	33.1	33.3	29.6	45.4	69.4	66.3	69.6	69.3		29.7	27.7	27.5	34.8	34.3	34.9	18.8	30.6	29.9	28.5
TENUI_European wheat striate mosaic virus_NC_078407	33	28.7	39	40.6	40.7	40.1	25.7	23.4	30.1	28.7	30	29.7	29.7	29.7		23	27.4	30.5	30.3	27.9	16	23.7	23.9	23.6
COGU_Watermelon crinkle leaf-associated virus 2_NC_079049	25.9	30.4	23.7	23.7	23.9	23	29.1	38.6	26.4	29.1	26.8	27.5	27.9	27.7	23		23.6	28.1	27.9	27.2	28.5	48.4	50	44.3
MECHLORO_Melon chlorotic spot virus_NC_040454	27.1	26.2	26.4	28.9	28.9	27.7	26.8	21.9	28.5	29.2	29.9	29	29.2	27.5	27.4	23.6		28.5	25.4	27.8	17.4	24.4	25.2	24.9
LAULA_Grapevine associated cogu-like virus 3_NC_078439	28.5	34.5	28.2	29	29	28.5	27.2	27	33.2	35	34.6	34.8	35	34.8	30.5	28.1	28.5		64.2	70.4	19.3	29.3	27.7	26.5
LAULA_Laurel Lake virus_NC_043681	28.1	33.2	28.2	29.1	30.1	29.1	25.7	26.8	33.4	34.2	35	34.7	34.7	34.3	30.3	27.9	25.4	64.2		64.2	17.9	28.2	28.2	26.5
LAULA_Grapevine associated cogu-like virus 2_NC_078436	28.3	32	29.2	27.7	28.6	28.7	26.5	28.2	34.2	35.2	33.3	33.9	33.9	34.9	27.9	27.2	27.8	70.4	64.2		19.1	28.6	27.6	27.7
COGU_Brassica campestris chinensis coguvirus 1_NC_079046	16.4	20.8	17.2	18.2	18.8	18.5	21	25.1	19.4	18.6	19.1	18.4	18.7	18.8	16	28.5	17.4	19.3	17.9	19.1		30.9	30	30.8
COGU_Watermelon crinkle leaf-associated virus 1_NC_079047	25.1	29.7	25.3	25.8	26.5	26.2	31	36.5	29.6	29.3	30.1	28.8	30.3	30.6	23.7	48.4	24.4	29.3	28.2	28.6	30.9		48.6	48
COGU_Citrus concave gum-associated virus_NC_035454	25.3	31.8	25	24.6	24.8	24.6	30.5	39.2	27.4	29.6	28.7	29.8	30	29.9	23.9	50	25.2	27.7	28.2	27.6	30	48.6		54.9
COGU_Citrus virus A_NC_078104	24.8	30.7	24	23.2	24.1	24.2	29.2	37.1	27.2	28.7	28.3	27	28	28.5	23.6	44.3	24.9	26.5	26.5	27.7	30.8	48	54.9	

F) MP (nt identities)

	guvirus_Grapevine associated cogu-like virus 1_NC_078434	virus_Brassica campestris chinensis coguvirus 1_NC_079046	virus_Watermelon crinkle leaf-associated virus 2_NC_079049	virus_Watermelon crinkle leaf-associated virus 1_NC_079047	Coguvirus Coguvirus eburi_Citrus virus A_NC_078104	virus citri_Citrus concave gum-associated virus_NC_035454	TENUI_Tenuivirus oryzalbae_NC_036599	TENUI_Tenuivirus urochloae_NC_038758	TENUI_Tenuivirus echinochloae_NC_038935	Phlebo_OUT_OR610328	**RUBOD_Raspberry_rubodvirus_1_MPb**	**RUBOD_Raspberry_rubodvirus_1_MPa**	LAULA_Grapevine associated cogu-like virus 4_NC_078447	LAULA_Grapevine associated cogu-like virus 3_NC_078438	LAULA_Grapevine associated cogu-like virus 2_NC_078435	RUBOD__Grapevine Muscat rose virus_NC_078403	RUBOD__Apple rubbery wood virus 2_NC_055537	RUBOD_Apple rubbery wood virus 1_NC_055391	RUBOD__Grapevine Garan dmak virus_NC_078406	RUBOD__Apple rubbery wood virus 2_NC_055536
Coguvirus Grapevine coguvirus_Grapevine associated cogu-like virus 1_NC_078434		28.9	28.4	25.9	28.6	28.4	16.7	17.1	16.6	18.3	19.2	22.6	21.2	23.2	23.4	21.9	23.6	22.2	23.5	22.5
Coguvirus_Brassica campestris chinensis coguvirus 1_NC_079046	28.9		43.4	41	44.4	43.6	18.8	18.5	18	19	21	25.4	19.7	21.1	20.9	22.8	25.6	24.7	25.9	24.9
Coguvirus_Watermelon crinkle leaf-associated virus 2_NC_079049	28.4	43.4		44	48.9	45.2	17.8	18.4	17.6	19.4	21	23.1	19	20.2	20.2	22.5	23.9	23.2	24.6	23.7
Coguvirus_Watermelon crinkle leaf-associated virus 1_NC_079047	25.9	41	44		47.3	45.9	18.4	19.2	18.5	20.2	22.2	23.6	19.1	19.4	19.5	23.4	24.4	23.4	25.4	24.6
Coguvirus Coguvirus eburi_Citrus virus A_NC_078104	28.6	44.4	48.9	47.3		58.8	19	18.7	18.1	20.2	21.8	24.4	19.3	20.3	19.7	25.8	25.4	25.3	25.7	25.7
Coguvirus Coguvirus citri_Citrus concave gum-associated virus_NC_035454	28.4	43.6	45.2	45.9	58.8		18.9	18	16.5	18.8	20.6	24.3	19.7	20.2	20.4	24.2	23.9	24.6	24.7	24.7
TENUI_Tenuivirus oryzalbae_NC_036599	16.7	18.8	17.8	18.4	19	18.9		80.2	80.4	21.4	19.1	21.8	14.4	16.6	17.3	25.1	26.5	25.9	26.9	28.1
TENUI_Tenuivirus urochloae_NC_038758	17.1	18.5	18.4	19.2	18.7	18	80.2		83.3	21.9	19.9	22.9	14.7	16.5	17.1	26.9	26.6	26.4	26.9	28.3
TENUI_Tenuivirus echinochloae_NC_038935	16.6	18	17.6	18.5	18.1	16.5	80.4	83.3		23.1	19.3	21.8	14.4	16.3	16.8	25.3	27	26.3	26.7	27.8
Phlebo_OUT_OR610328	18.3	19	19.4	20.2	20.2	18.8	21.4	21.9	23.1		28.5	23.6	18.4	21.4	22.4	29.7	30.9	29.4	33.2	32.5
**RUBOD_Raspberry_rubodvirus_1_MPb**	19.2	21	21	22.2	21.8	20.6	19.1	19.9	19.3	28.5		30.1	18.7	21.4	21.4	35	35.5	34.5	35.2	36.3
**RUBOD_Raspberry_rubodvirus_1_MPa**	22.6	25.4	23.1	23.6	24.4	24.3	21.8	22.9	21.8	23.6	30.1		24.8	26.4	26.4	41.1	41	41.2	43.3	42.5
LAULA_Grapevine associated cogu-like virus 4_NC_078447	21.2	19.7	19	19.1	19.3	19.7	14.4	14.7	14.4	18.4	18.7	24.8		52.9	53.7	22.5	23.5	22.2	24.1	23.3
LAULA_Grapevine associated cogu-like virus 3_NC_078438	23.2	21.1	20.2	19.4	20.3	20.2	16.6	16.5	16.3	21.4	21.4	26.4	52.9		84.2	27.2	28.5	26.9	28.1	27.5
LAULA_Grapevine associated cogu-like virus 2_NC_078435	23.4	20.9	20.2	19.5	19.7	20.4	17.3	17.1	16.8	22.4	21.4	26.4	53.7	84.2		26.4	28.9	27.5	29	28
RUBOD__Grapevine Muscat rose virus_NC_078403	21.9	22.8	22.5	23.4	25.8	24.2	25.1	26.9	25.3	29.7	35	41.1	22.5	27.2	26.4		60.3	59.2	61.4	65.1
RUBOD__Apple rubbery wood virus 2_NC_055537	23.6	25.6	23.9	24.4	25.4	23.9	26.5	26.6	27	30.9	35.5	41	23.5	28.5	28.9	60.3		61	65.1	65.2
RUBOD_Apple rubbery wood virus 1_NC_055391	22.2	24.7	23.2	23.4	25.3	24.6	25.9	26.4	26.3	29.4	34.5	41.2	22.2	26.9	27.5	59.2	61		61.2	63.7
RUBOD__Grapevine Garan dmak virus_NC_078406	23.5	25.9	24.6	25.4	25.7	24.7	26.9	26.9	26.7	33.2	35.2	43.3	24.1	28.1	29	61.4	65.1	61.2		79.8
RUBOD__Apple rubbery wood virus 2_NC_055536	22.5	24.9	23.7	24.6	25.7	24.7	28.1	28.3	27.8	32.5	36.3	42.5	23.3	27.5	28	65.1	65.2	63.7	79.8	
